# Transcriptional and pathway analysis in the hypothalamus of newly hatched chicks during fasting and delayed feeding

**DOI:** 10.1186/1471-2164-11-162

**Published:** 2010-03-09

**Authors:** Stacy E Higgins, Laura E Ellestad, Nares Trakooljul, Fiona McCarthy, Jason Saliba, Larry A Cogburn, Tom E Porter

**Affiliations:** 1 Department of Animal and Avian Sciences, University of Maryland, College Park, MD 20742, USA; 2 Animal and Food Sciences Department, University of Delaware, Newark, DE 19716, USA; 3 Department of Basic Sciences, College of Veterinary Medicine, Mississippi State University, Starkville, MS 39762, USA

## Abstract

**Background:**

The hypothalamus plays a central role in regulating appetite and metabolism. However, the gene networks within the hypothalamus that regulate feed intake and metabolism, and the effects of fasting on those pathways are not completely understood in any species. The present experiment evaluated global hypothalamic gene expression in newly hatched chicks using microarray analysis to elucidate genes and pathways regulated by feeding, fasting, and delayed feeding. Ten groups of chicks were sampled over four days post-hatch, including fed, fasted, and 48 h fasted followed by access to feed for 4 h, 24 h, and 48 h. Hypothalamic samples were collected for microarray analysis (n = 4). Expression patterns of selected genes were confirmed by quantitative real-time PCR. Pathway analysis of the microarray results predicted a network of genes involved in neuropeptide or neurotransmitter signaling. To confirm the functionality of this predicted gene network, hypothalamic neurons from fed and fasted chicks were isolated and cultured in the presence of neuropeptide Y, somatostatin, α-melanocyte stimulating hormone, norepinephrine, and L-phospho-serine. Results confirmed functional relationships among members of the predicted gene network. Moreover, the effects observed were dependant upon the nutritional state of the animals (fed *vs*. fasted).

**Results:**

Differences in gene expression (≥ 1.6 fold) were detected in 1,272 genes between treatments, and of those, 119 genes were significantly (P < 0.05) different. Pathway Miner analysis revealed that six genes (*SSTR5*, *NPY5R*, *POMC*, *ADRB2*, *GRM8*, and *RLN3*) were associated within a gene network. *In vitro *experiments with primary hypothalamic neurons confirmed that receptor agonists involved in this network regulated expression of other genes in the predicted network, and this regulation within the network was influenced by the nutritional status and age of the chick.

**Conclusions:**

Microarray analysis of the hypothalamus during different nutritional states revealed that many genes are differentially regulated. We found that functional interactions exist among six differentially regulated genes associated within a putative gene network from this experiment. Considering that *POMC*, an important gene in controlling metabolism, was central to this network, this gene network may play an important role in regulation of feeding and metabolism in birds.

## Background

The hypothalamus regulates feed intake and satiety in vertebrates. However, species differ greatly in their feeding habits. Intensive genetic selection for growth rate of broiler (meat-type) chickens has resulted in a remarkable increase in feed intake. Since the hypothalamus regulates feed intake and satiety in vertebrates, the function of the chicken hypothalamus has been extensively studied in response to numerous hormones and neuropeptides [1]. Importantly, hypothalamic function of chickens is well correlated with other species in many respects [2-4]. A few exceptions include the lack of response in the chicken to specific orexigenic peptides, such as orexin-A and B, galanin, motilin, and melanin concentrating hormone [3]. Despite the information available, the specific mechanisms by which the hypothalamus coordinates all inputs to control feeding and metabolism in chickens, or any other avian species, is not understood, as it is difficult to evaluate all possible changes within the animal simultaneously. Similarly, a comprehensive analysis of hypothalamic gene expression related to appetite and satiety has not been reported for any species.

Microarrays have been recently used to evaluate genes within the liver of 4-week-old chickens that were fed or fasted [5]. In that study, many gene expression differences directly regulating metabolism and nutrient utilization were revealed, and it was determined that fasting, in general, caused more down-regulation of genes than up-regulation. Specifically, they observed that 2,062 genes were differentially regulated by fasting. In the present study, we utilized microarrays to examine global gene expression in the hypothalamus during feeding, fasting, or delayed feeding of newly hatched chicks. Since the hypothalamus integrates hunger and satiety input from the digestive tract, liver, and fat stores, we examined the transcriptional response of the hypothalamus to nutrient deprivation. Evaluation of global gene expression in the hypothalamus during fasting or after delayed feeding allowed us to evaluate genes known to be associated with feed intake and metabolism, as well the opportunity to discover genes not previously associated with the regulation of feed intake and metabolism. Hypothalamic neurons isolated from fed and fasted chicks were then treated in culture to evaluate the functionality of one gene network predicted from the microarray results.

## Results

### Experiment 1 Microarray and qRT-PCR Analysis

These experiments were performed to analyze global gene expression in the hypothalamus of newly hatched chicks fasted, fed, or fed following a 48 h fast. A description of all treatment groups is detailed in Table 1. Body weight and yolk sac weight were obtained at each sampling time (Table 2). Mean body weight at hatch was 40 g, and this did not significantly change within 24 hours in fed or fasted chicks. However, by 48 h, fed chicks were significantly heavier than chicks fasted or delayed fed for 4 h. At 72 h and 96 h, fed chicks were again heavier (p < 0.05) than their delayed fed counterparts. Remarkably, by 96 h, fed chicks had effectively doubled their hatching body weight. Yolk sac weights were not significantly different between treatments within a time point, and steadily decreased with increasing age of the chicks.

**Table 1:** Description of full fed (FF), fasted (NF), and delayed fed (DF) treatments administered to chicks.

Abbreviation	Fasted	Fed	Time of Sample Collection
H			Within 4 h of hatch
24FF		24 h Fully Fed	24 h
24NF	24 h Not Fed		24 h
48FF		48 h Fully Fed	48 h
48NF	48 h Not Fed		48 h
4DF	48 h Not Fed	4 h Delayed Fed	52 h
72FF		72 h Fully Fed	72 h
24DF	48 h Not Fed	24 h Delayed Fed	72 h
96FF		96 h Fully Fed	96 h
48DF	48 h Not Fed	48 h Delayed Fed	96 h

**Table 2:** Body weight and yolk sac weight in full fed (FF), fasted (NF) and delayed fed (DF) chicks.

Treatment	Body Weight (g)	Yolk Sac Weight (g)
H	40.15 ± 1.5^f^	4.66 ± 0.2^a^
24FF	42.12 ± 1.5^ef^	2.64 ± 0.2^b^
24NF	39.11 ± 1.5^f^	3.14 ± 0.2^b^
48FF	58.27 ± 1.5^d^	1.60 ± 0.2^cd^
48NF	37.90 ± 1.5^f^	1.77 ± 0.2^cd^
4DF	43.44 ± 1.5^ef^	1.47 ± 0.2^cd^
72FF	74.13 ± 1.5^b^	0.98 ± 0.2^de^
24DF	53.17 ± 1.5^ef^	0.81 ± 0.2^e^
96FF	95.77 ± 1.5^a^	0.56 ± 0.2^e^
48DF	66.09 ± 1.5^c^	0.55 ± 0.2^e^

Differing letters within columns indicate significant (p < 0.05) differences.

Microarray analysis was performed using the Operon/ARK 20.7 K long oligo arrays, which are printed with 21,120 chicken oligo probes. Data were deposited in the NCBI Gene Expression Omnibus, accession number GSE13257 http://www.ncbi.nlm.nih.gov/geo/query/acc.cgi?acc=GSE13257. Our analysis returned data for 5,855 probes on at least 3 out of 4 slides within a group. Of these, 1,272 differed in expression levels by at least 1.6 fold among treatment groups. Among the 1,272 genes that differed in expression levels, 119 were significantly different (P < 0.05) as determined by ANOVA. Of these, 60 were shown to be upregulated in fasted chicks at 48 h as compared to fed chicks (Additional File 1, Table S1), and 59 genes were downregulated in fasted compared to fed chicks at 48 h (Additional File 2, Table S2). Additionally, the 119 genes found to be differentially regulated were functionally characterized using GO terms, and these terms are included in Additional File 3, Table S3. Evaluation of the biological process GO terms for these genes revealed that many different biological processes in the hypothalamus are affected by fasting of chicks, as there were no terms encompassing the majority of genes.

**Figure 1:**
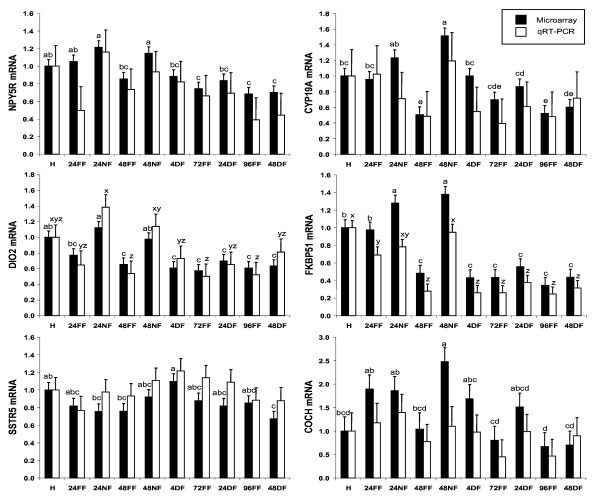
**Relative levels of mRNA for selected genes upregulated by fasting as determined by microarray and qRT-PCR**. Data were normalized to the housekeeping gene phosphoglycerate kinase 1 (*PGK1*), and are expressed relative to mRNA levels at hatch, which are set to the value of 1. Gene expression higher than 1 indicates upregulation compared to hatch, while gene expression levels less than 1 indicate downregulation compared to hatch. Chicks were either fully fed (FF), not fed (NF) or delayed fed (DF). Treatment groups are designated as follows: hatch (H), fed for 24 h (24FF), fasted for 24 h (24NF), fed for 48 h (48FF), fasted for 48 h (48NF), fasted for 48 h then fed for 4 h (4DF), fed for 72 h (72FF), fasted for 48 h then fed for 24 h (24DF), fed for 96 h (96FF), fasted for 48 h then fed for 48 h (48DF). Genes evaluated were: neuropeptide Y receptor 5 (*NPY5R*), aromatase (*CYP19A*), deiodinase II (*DIO2*), FK506 binding protein 51 (*FKBP51*), somatostatin receptor 5 (*SSTR5*), and coagulation factor C (*COCH*). Black bars are microarray results, and white bars are qRT-PCR results. Values within a single method with differing letters are significantly different (*P *< 0.05).

**Figure 2:**
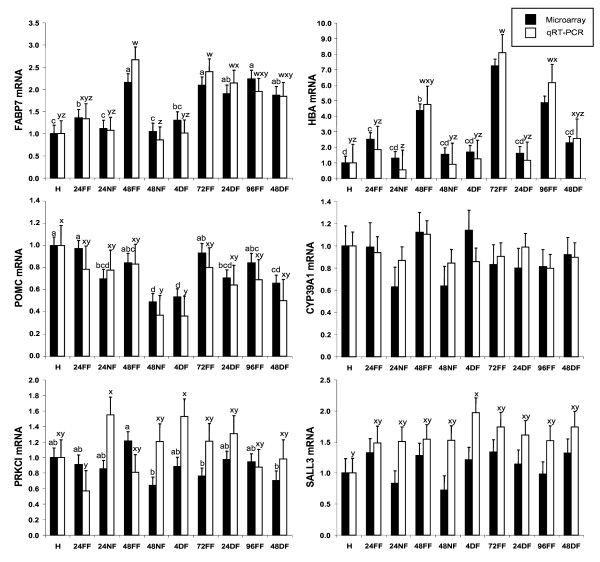
**Relative levels of mRNA for selected genes downregulated by fasting as determined by microarray and qRT-PCR**. Data were normalized to the housekeeping gene phosphoglycerate kinase 1 (*PGK1*), and are expressed relative to mRNA levels at hatch, which are set to the value of 1. Gene expression higher than 1 indicates upregulation compared to hatch, while gene expression levels less than 1 indicate downregulation compared to hatch. Treatment groups are designated as follows: hatch (H), fed for 24 h (24FF), fasted for 24 h (24NF), fed for 48 h (48FF), fasted for 48 h (48NF), fasted for 48 h then fed for 4 h (4DF), fed for 72 h (72FF), fasted for 48 h then fed for 24 h (24DF), fed for 96 h (96FF), fasted for 48 h then fed for 48 h (48DF). Genes evaluated were: fatty acid binding protein 7 (*FABP7*), hemoglobin alpha (*HBA*), pro-opiomelanocortin (*POMC*), oxysterol 7-alpha-hydroxylase (*CYP39A1*), protein kinase C iota (*PRKCI*), and sal-like 3 (*SALL3*). Black bars are microarray results, and white bars are qRT-PCR results. Values within a single method with differing letters are significantly different (P < 0.05).

RNA samples were analyzed by qRT-PCR to confirm differential expression of 12 genes found to be significantly affected by fasting with the microarray analysis (Figures 1 and 2). Selected genes for qRT-PCR that were upregulated by fasting for 24 or 48 h included neuropeptide Y receptor 5 (*NPY5R*), deiodinase II (*DIO2*), somatostatin receptor 5 (*SSTR5*), aromatase (*CYP19A1*), FK506 binding protein 51 (*FKBP51*), and coagulation factor C (*COCH*) (Figure 1). Selected downregulated genes were fatty acid binding protein 7 (*FABP7*), pro-opiomelanocortin (*POMC*), and protein kinase C iota (*PRCKI*). Additional genes found to be downregulated by fasting in the microarray were hemoglobin alpha (*HBA*), oxysterol 7-α-hydroxylase-2 (*CYP39A*) and sal-like 3 (*SALL3*). The genes most highly upregulated by fasting in chicks were *CYP19A *and *FKBP51*, which exhibited a 3-fold increase in expression due to fasting at 48 h (Figure 1). Five of the six genes that were up-regulated in the 48 h fasted group returned to fed levels after 4 h of feeding, and *CYP19A1 *returned to fed levels by 24 h of feeding. Of the down-regulated genes, *HBA *exhibited a 5-fold decrease in expression in fasted chicks at 48 h, and *FABP7 *exhibited a 3-fold decrease in expression in fasted chicks (Figure 2). In general, genes down-regulated by fasting required delayed feeding of at least 24 h to return to fed levels, with the exception of *HBA*, which did not return to the levels of fed chicks by 48 h of delayed feeding.

**Figure 3:**
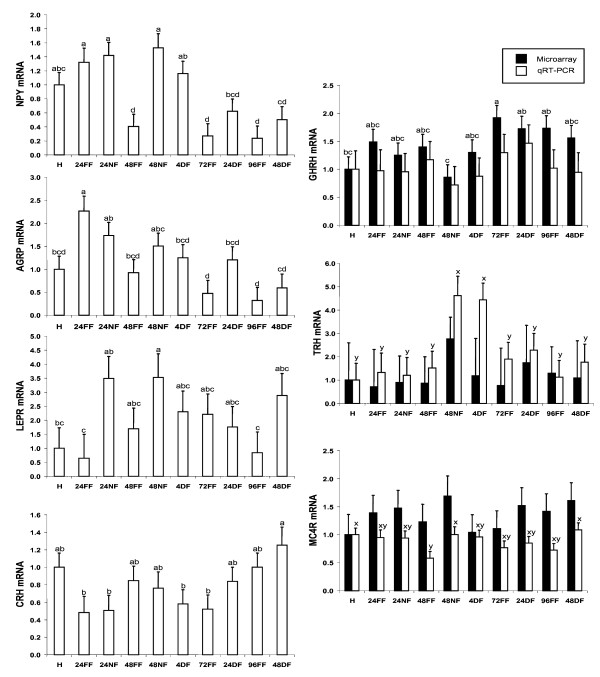
**Relative levels of mRNA for selected genes associated with feeding and fasting in the hypothalamus as determined by microarray and qRT-PCR**. Data were normalized to the housekeeping gene phosphoglycerate kinase 1 (*PGK1*), and are expressed relative to mRNA levels at hatch, which are set to the value of 1. Gene expression higher than 1 indicates upregulation compared to hatch, while gene expression levels less than 1 indicate downregulation compared to hatch. Treatment groups are designated as follows: hatch (H), fed for 24 h (24FF), fasted for 24 h (24NF), fed for 48 h (48FF), fasted for 48 h (48NF), fasted for 48 h then fed for 4 h (4DF), fed for 72 h (72FF), fasted for 48 h then fed for 24 h (24DF), fed for 96 h (96FF), fasted for 48 h then fed for 48 h (48DF). Genes evaluated were neuropeptide Y (*NPY*), agouti-related protein (*AGRP*), leptin receptor (*LEPR*), corticotropin releasing hormone (*CRH*), growth hormone releasing hormone (*GHRH*), thyrotropin releasing hormone (*TRH*), and melanocortin receptor 4 (*MC4R*). Black bars are microarray results, and white bars are qRT-PCR results. Values within a single method with differing letters are significantly different (P < 0.05).

We also performed qRT-PCR on genes of interest in the hypothalamus that have been associated with feeding and fasting, metabolism, and growth. These genes were either not represented on the microarray or did not meet the criteria for candidate genes (either too few data points, fold changes less than 1.6, or not significant by ANOVA). These genes included neuropeptide Y (*NPY*), agouti-related protein (*AGRP*), leptin receptor (*LEPR*), corticotropin releasing hormone (*CRH*), growth hormone releasing hormone (*GHRH*), thyrotropin releasing hormone (*TRH*) and melanocortin 4 receptor (*MC4R*) (Figure 3). As expected, *NPY *and *TRH *had significantly increased expression after 48 h of fasting, and mRNA levels did not return to the level of fed chicks until 24 h of delayed feeding. *MC4R *was moderately and significantly upregulated in chicks fasted for 48 h. *LEPR *exhibited increased expression in chicks fasted 24 h. *AGRP *likewise showed a non-significant increase in expression after 48 h of fasting. *GHRH *and *CRH *did not differ significantly in expression levels between treatments at a single time point.

### Experiment 1 Pathway Analysis

**Figure 4:**
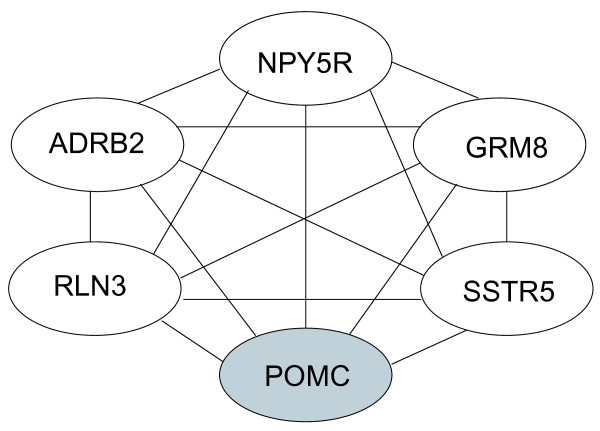
**Diagram of associated genes derived from the online Pathway Miner tool for clustering genes associated within a pathway**. Associated genes are neuropeptide Y receptor 5 (*NPY5R*), metabotropic glutamate receptor 8 (*GRM8*), somatostatin receptor 5 (*SSTR5*), pro-opiomelanocortin (*POMC*), relaxin 3 (*RLN3*), and beta-2-adrenergic receptor (*ADRB2*). Gene expression data generated by the microarray revealed that *POMC *exhibited reduced expression in fasted chicks, and the other associated genes were expressed at increased levels in fasted chicks.

Gene IDs for human orthologs of 87 differentially expressed genes were submitted to Pathway Miner, an online tool which searches three publicly available databases and determines gene interaction networks or pathways represented in the list of genes submitted. Analysis of our candidate genes revealed an interaction network of six genes (Figure 4): *NPY5R*, metabotropic glutamate receptor 8 (*GRM8*), *SSTR5*, *POMC*, relaxin 3 (*RLN3*) and beta-2-adrenergic receptor (*ADRB2*). Expression of all genes increased in response to fasting with the exception of *POMC*, which was reduced by fasting.

Gene networks were then analyzed with Ingenuity Pathway Analysis software. Analysis of genes significantly regulated between fed and fasted chicks on d1 and d2 with a fold difference of ≥ 1.4 revealed two primary pathways (Additional Files 4 and 5, Figure S1 and S2). Further analysis using a dataset of 119 genes differentially regulated between any two groups with a fold difference ≥ 1.6 revealed a putative pathway that included the genes *POMC*, *ADRB2*, and *SSTR5*, which were also constituents of the Pathway Miner output (Additional File 6, Figure S3).

### Experiment 2

**Figure 5:**
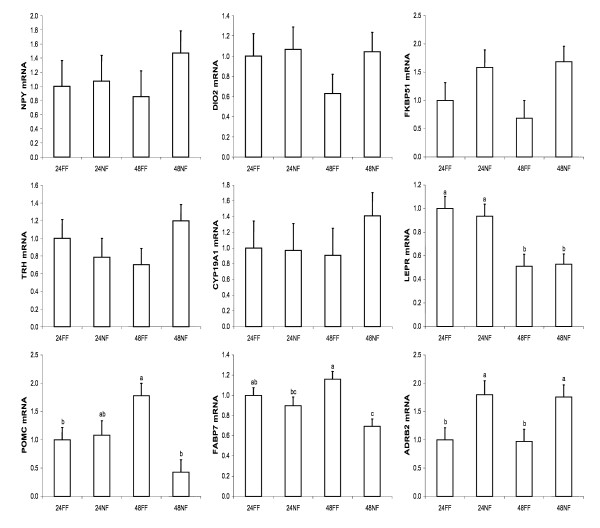
**Relative levels of mRNA determined by qRT-PCR for selected genes associated with feeding and fasting in the hypothalamus**. Data were normalized to the housekeeping gene phosphoglycerate kinase 1 (PGK1), and are expressed relative to mRNA levels in the 24 h fed group (24FF). Treatment groups are designated as follows: fed for 24 h (24FF), fasted for 24 h (24NF), fed for 48 h (48FF), and fasted for 48 h (48NF). Values with differing letters are significantly different (P < 0.05). Genes evaluated were: neuropeptide Y (NPY), deiodinase II (DIO2), FK506 binding protein 51 (FKBP51), thyrotropin releasing hormone (TRH), aromatase (CYP19A1), leptin receptor (LEPR), pro-opiomelanocortin (POMC), fatty acid binding protein 7 (FABP7), and beta-2-adrenergic receptor (ADRB2).

In order to confirm the effects of fasting on differential expression of selected genes, we performed another experiment in which chicks were again hatched and fed or fasted for 24 or 48 h. qRT-PCR was utilized to determine mRNA levels of *NPY*, *DIO2*, *FKBP51*, *TRH*, *CYP19A1*, *LEPR*, *POMC*, *FABP7*, and *ADRB2 *(Figure 5). In general, expression in fasted compared with fed birds was consistent with the results from Experiment 1. *POMC *and *FABP7 *were significantly reduced in fasted chicks at 48 h. *LEPR *was expressed at significantly lower levels at 48 h compared with 24 h, regardless of feeding status. Expression of *NPY*, *DIO2*, *FKBP51*, *TRH*, and *CYP19A1 *did not significantly differ in expression levels between treatments, yet the trend of increased expression of these genes in the 48NF group was consistent with the expression data from Experiment 1. This reduced number of significant effects in Experiment 2 may be attributed to the fewer number of samples utilized in the second experiment (n = 4 vs. n = 8 for Exp. 1). *MC4R*, *SSTR5*, and *NPY5R *were also evaluated (data not shown) and did not change due to treatment. *ADRB2 *was evaluated due to its presence in the proposed pathway. ADRB2 mRNA was significantly increased in chicks fasted for both 24 and 48 h, which was consistent with the microarray expression analysis (data not shown).

### Experiment 3 Culture and treatment of primary hypothalamic neurons

To determine whether the gene network predicted by both Pathway Miner and Ingenuity Pathway Analysis was indeed functional, we isolated primary hypothalamic neurons from chicks following fasting or feeding for 24 or 48 h, and treated them with hormones or receptor agonists *in vitro*. Our goal was to determine if the genes within the network could be regulated by treatments corresponding to receptors within this gene network. This experiment also evaluated the effects of nutritional status of the chicks (fed or fasted) and timing (24 or 48 h) on the effects of treatments *in vitro*. Following treatments in culture, gene expression was determined by qRT-PCR.

**Figure 6:**
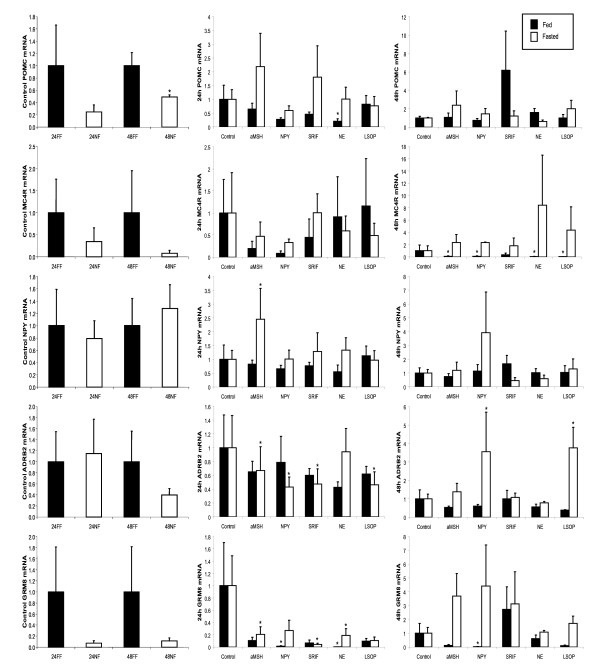
**Gene expression in dispersed hypothalamic neurons isolated from chicks following feeding or fasting for 24 or 48 h and treated with receptor agonists in culture**. Following feeding or fasting of chicks for 24 or 48 h, the hypothalamus was collected and neurons were dispersed and cultured in 12-well culture dishes in neurobasal medium for 24 h. Receptor agonists [α- melanocyte stimulating hormone (aMSH), neuropeptide Y (NPY), somatostatin (SRIF), norepinephrine (NE), or O-phospho-L-serine (LSOP, a group III metabotropic receptor agonist] were added to the medium following 24 h of incubation, and neurons were incubated for an additional 24 h. Cells were collected, and RNA was extracted for analysis by qRT-PCR. Gene expression was evaluated for pro-opiomelanocortin (*POMC*), melanocortin 4 receptor (*MC4R*), neuropeptide Y (*NPY*), beta-2-adrenergic receptor (*ADRB2*), and metabotropic glutamate receptor 8 (*GRM8*). The first column compares the relative gene expression levels between fed and fasted chicks within each time point (24 h - white bars, 48 h - black bars). Gene expression levels in treated neurons from chicks fed (black bars) or fasted (white bars) for 24 h are represented in the 2nd column, and levels from chicks fed (black bars) or fasted (white bars) for 48 h are in the 3rd column. Asterisks indicate significant differences (P < 0.05) within time points for the control graphs (column 1) or within time and nutritional status (fed or fasted) (columns 2 and 3).

**Figure 7:**
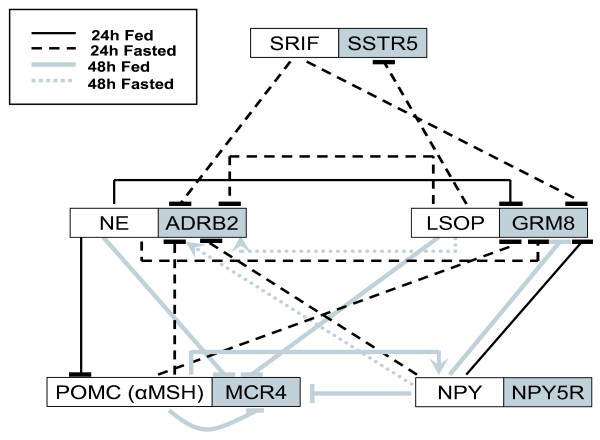
**Summary of changes in gene expression of primary hypothalamic neurons obtained from chicks following feeding or fasting for 24 or 48 h and treated in cell culture**. Gray boxes represent receptors and white boxes represent the receptor agonists utilized for treatment in culture. Lines indicate a significant effect on a specific gene following addition of the agonist in culture as compared with the non-treated control within the same group (fed or fasted) and time point (24 or 48 h). Lines ending in arrows indicate an increase in gene expression level, and lines ending with a bar indicate a reduction in gene expression level due to treatment.

Primary hypothalamic neurons were isolated from chicks fed or fasted for 24 or 48 h, and maintained in culture in neurobasal medium (NBM) for 24 h. Treatments were then added to the medium for 24 h. Treatments included alpha-melanocyte stimulating hormone (αMSH), which is the primary hormone derived from POMC in the hypothalamus; neuropeptide Y (NPY), the hormone agonist for NPY5R; somatostatin (SRIF), the hormone agonist for SSTR5; nor-epinephrine (NE) an agonist for ADRB2; and LSOP which functions as a type III metabotropic receptor agonist, of which GRM8 is a member. Total RNA was isolated and gene expression determined using qRT-PCR for the following genes: *POMC, MC4R, NPY, NPYR5, ADRB2, GRM8*, and *SSTR5*. Gene expression in non-treated control wells was significantly different only between 48 h fed and fasted chicks for *POMC*, which correlated with data from the microarray where POMC was likewise decreased by fasting (Figure 6). Three genes were significantly regulated by multiple treatments added to the medium. *MC4R *expression was reduced by all treatments in fed chicks at 48 h. *ADRB2 *was significantly reduced by αMSH, NPY, SRIF, and LSOP in fasted chicks at 24 h, but increased by NPY and LSOP in fasted chicks at 48 h. Expression of *GRM8 *at 48 h was regulated by αMSH, NPY, and LSOP in such a way that it was downregulated in fed chicks and increased (though not significantly so) in fasted chicks. Gene expression was also measured for *NPY5R *and *SSTR5 *(data not shown), with the only significant differences being reduced expression of *SSTR5 *in 48 h fasted controls compared with 48 h fed controls, and reduced expression of *SSTR5 *in the presence of LSOP in 24 h fasted chicks. A summary of significant differences due to treatment is shown in Figure 7.

## Discussion

The hypothalamus controls feeding and satiety in vertebrates, and directs growth and metabolism [6]. We performed microarray analysis to evaluate global gene expression in the hypothalamus in order to identify genes and gene networks differentially expressed in response to fasting and feeding of newly hatched chicks. Our analysis compared gene expression in the hypothalamus of chicks that were fed or fasted for 24 or 48 h, as well as chicks that were fed following a 48 h fast. Fasting of chicks can retard growth compared to chicks fed immediately for up to 6 weeks of age [7], yet the mechanisms behind the long-term effects of fasting on growth and metabolism are not known. Therefore, we included delayed feeding treatments (fasted for 48 h, then fed for 4 h, 24 h, or 48 h) to provide insight into potential gene expression differences that may persist after animals are allowed access to feed.

Among genes that were increased by fasting, were the neuropeptide receptors *NPY5R *and *SSTR5*. As NPY is known to be increased in fasted animals [8], and intra-cerebroventricular (icv) administration of this neuropeptide causes increased feed intake [9], the upregulation of its receptor due to fasting is not surprising. SSTR5 is a somatostatin receptor that is primarily found in the pituitary gland. However, *SSTR5 *mRNA in the central nervous system of rats was detected primarily in the hypothalamus and preoptic area [10]. Receptor subtype-specific immuno-histochemistry of the rat hypothalamus revealed few SSTR5-positive cells, and those that were observed were primarily found in the median eminence [11]. The role that SSTR5 plays in the hypothalamus is not clear.

*DIO2 *expression was significantly increased in fasted chicks at both 24 and 48 h, returning immediately to the levels of fed chicks in the 4DF group. This was a particularly interesting finding, because DIO2 converts thyroid hormones from inactive T_4 _to active T_3_, and we anticipated an opposite effect of fasting. However, elevated levels of DIO2 activity would be expected to cause increased T_3 _within the hypothalamus, resulting in reduced TRH release through negative feedback [6]. In response, the pituitary gland would be expected to decrease release of TSH. Subsequently, levels of circulating T_3 _would then be reduced, and the metabolic rate of the chick would be reduced, ultimately in response to the elevated hypothalamic DIO2. Thus, it could be predicted that increased hypothalamic DIO2 activity would ultimately decrease metabolism through feedback mechanisms. The increased *DIO2 *mRNA levels observed in response to fasting are consistent with this model. Other genes significantly increased by fasting for 48 h include *CYP19A1 *(aromatase), *FKBP51*, and *COCH*. Their respective functions include conversion of androgens to estrogen, facilitating translocation of proteins such as nuclear hormone receptors to the nucleus, and providing structure to the ear. The significance of increased expression of these genes within the hypothalamus in fasted chicks is unknown.

Among genes with reduced expression due to fasting were the genes *FABP7*, which is a brain-specific fatty acid binding protein, *POMC *(which encodes for a precursor polypeptide to multiple hormones including αMSH), and *PRKCI*. Importantly, hypothalamic αMSH is known to increase metabolic rate. Therefore, it was not surprising to see *POMC *mRNA decreased by fasting, presumably as part of the overall mechanism down-regulating metabolism to compensate for decreased feed intake. All genes reduced by fasting returned to similar expression levels as full-fed chicks within 24 h of delayed feeding.

*CYP39A1 *and *SALL3 *genes were also down-regulated by fasting at 24 and 48 h. CYP39A1 functions primarily to convert cholesterol to bile acids, and SALL3 is a transcription factor. Information on the function of these genes specifically in chickens was not available. Further, the qRT-PCR expression results for both genes were not completely correlated with the microarray results. We also observed a significant reduction in expression of *HBA *in the hypothalamus of fasted chicks, which did not return to the levels of fed chicks even after 48 h of delayed feeding. The specific function and potential importance of the reduction of HBA in the hypothalamus of chicks due to fasting is not known.

Seven genes that are known to be associated with feed intake, metabolism or growth, for which microarray data were unavailable or the microarray analysis did not indicate significant differences, were also evaluated by qRT-PCR. These included genes for two orexigenic neuropeptides, *NPY *and *AGRP*. These two genes are co-expressed in neurons within the ARC that participate in the melanocortin pathway [12], and both were increased in the 48NF group. Increased *NPY *mRNA would be consistent with increased appetite in fasted birds, and increased AGRP, an antagonist to α MSH for MC4R, would be consistent with reduced metabolic rate in fasted chicks. We likewise observed a significant increase in expression of both *MC4R *and *TRH *mRNA in chicks fasted for 48 h, and a significant increase in *LEPR *expression was observed in 24NF chicks compared with 24FF chicks. Increased levels of hypothalamic *MC4R *and *LEPR *expression might be due to depressed levels of α MSH and leptin release in fasted animals. Interestingly, *TRH *mRNA levels were increased at 48 hr but not at 24 hr, which is consistent with reduced negative feedback from reduced circulating T_3 _levels associated with a reduction of metabolic rate in fasted birds.

Both NPY and AGRP can increase feeding in animals and inhibit the thyroid axis when administered icv, however they signal through different receptors [9,13,14]. NPY signals through NPY receptors, of which receptors Y1 and Y5 are associated with the induction of hyperphagia [15]. However, AGRP is an inverse agonist of MC4R, and therefore directly plays a part in the melanocortin pathway [16]. In fasted broiler (meat-type) chickens, AGRP returned feed intake to control levels when co-administered with αMSH, which inhibited feed intake alone. Interestingly, administration of AGRP alone to non-fasted broiler chickens or leghorn (egg laying-type) chickens only increased feed intake in the leghorn chickens [17].

Leptin is a hormone produced primarily by adipocytes in mammals, and high levels of leptin are generally indicative of satiety in mammals [18]. In this experiment, we observed an increase in leptin receptor (*LEPR*) mRNA levels in the hypothalamus following 24 h of fasting. Although we did not determine the levels of circulating leptin in this experiment, administration of synthetic leptin has been reported to reduce feeding in chickens [19], and these data could indicate that depressed leptin during fasting regulates expression of LEPR. Importantly, leptin is also a key regulator of the melanocortin system in the hypothalamus in mammals. Leptin has been shown to directly inhibit NPY and AGRP neurons and to stimulate POMC and CART neurons in the ARC of the hypothalamus in mice [20]. Although substantial controversy exists regarding the sequence of chicken leptin cDNA and peptide [21,22], the leptin receptor in chickens has been sequenced unequivocally [23,24], and chickens respond to recombinant chicken leptin as well as leptin derived from other species.

Further analysis using Pathway Miner software indicated that our candidate gene list was enriched in genes participating in a network of associated genes due to their ability to regulate cAMP production in cells. Genes from our list included in that network were: *NPY5R*, *GRM8*, *SSTR5*, *POMC*, *RLN3*, and *ADRB2 *(Figure 4). These genes represent hormones (RLN3 and POMC) and G-protein coupled receptors. The four receptors include two neuropeptide receptors (SSTR5 and NPY5R) and two amino-acid derived neurotransmitter (ADRB2 and GRM8) receptors. Among these genes, only *POMC *was downregulated by fasting in chicks, the other five genes were increased by fasting. As *POMC *encodes for the precursor of α MSH, an important regulator of metabolic rate, this finding raised the intriguing possibility that control of metabolism by the hypothalamus involves interactions between NPY, adrenergic, glutaminergic, and melanotropic neurons. Interestingly, *POMC*, *ADRB2*, and *SSTR5 *were also in a network predicted using Ingenuity Pathway Analysis. However, this network for gene interactions within the hypothalamus was only hypothetical, so further experiments were designed to test their putative interactions.

In order to test the functionality of this gene network, we performed another experiment *in vitro *with isolated primary hypothalamic neurons. We treated primary hypothalamic neurons with hormones or receptor agonists *in vitro *corresponding to the genes within the putative network. Our cell culture results confirmed that the gene network predicted from the microarray analysis is functional within the hypothalamus. Genes within the network can directly affect expression of other genes in the network. For example, *POMC *expression was decreased by treatment with NE, an agonist for ADRB2. Importantly, the activity and connectivity of this network was altered by the fed or fasted state of the chicks. This finding demonstrates that the metabolic perturbation of fasting was a useful tool in identifying genes that can control important regulators of metabolism, such as *NPY*, *POMC*, *MC4R*, *NPYR5*, *ADRB2*, and *GRM8 *(Figures 6 and 7). Beyond this, the specific mechanism by which they regulate gene expression during feeding and fasting of chicks was not determined. It should be noted that we were unable to obtain chicken relaxin 3 for evaluation in this experiment, though it was included as part of the network by Pathway Miner. Interestingly, the literature suggests that RLN3 is involved with regulation of feeding and satiety, as it does increase feeding in rats following icv or iPVN injection [25,26].

*SSTR5 *expression was changed little by treatments of cultured neurons, with reduced expression occurring only in the presence of LSOP in neurons from 24 h fasted chicks. In contrast, both *ADRB2 *and *GRM8 *expression were decreased by addition of SRIF to the culture medium. As *SSTR5 *was differentially regulated between fasted and fed groups *in vivo*, the relative lack of regulation of this receptor may indicate it is not regulated by other gene products in this network of genes. Alternatively, it may be regulated by other neurotransmitters, hormones, or metabolites affected by feeding or fasting. It should be noted, however, that SRIF treatment altered mRNA levels for *ADRB2*, *GRM8*, and *MC4R*, demonstrating a potential role for hypothalamic SRIF and SSTR5 in regulating metabolism.

NPY, the ligand for NPYR5, is a neuropeptide associated with increased hunger, and icv injection of NPY markedly increases feeding behavior in chickens [9] and other species [14]. NPY is produced in the arcuate nucleus of the hypothalamus, and co-expressed in neurons producing AGRP, another orexigenic peptide. Notably, although *NPYR5 *was identified as part of this putative pathway, no agonists in the cell culture were capable of modifying *NPYR5 *expression. However, NPY treatment suppressed levels of *MC4R *and *GRM8 *mRNA, consistent with a role for NPY in decreasing metabolic rate. Treatment with αMSH increased *NPY *expression in neurons from the hypothalamus of chicks fed for 48 h, suggesting the possibility of a negative feedback loop within the hypothalamus controlling metabolism. Interestingly, icv administration of NPY and αMSH reduced feed intake in chicks at the same level as administration of αMSH alone [27], suggesting that melanocortin has a greater effect on feed intake than NPY in chickens.

*POMC *is a principle component of the melanocortin system, which is centered in the arcuate nucleus of the hypothalamus (known as the infundibular nucleus in chickens) [28,29]. POMC is a precursor polypeptide which is cleaved into ten individual hormones, including αMSH. The melanocortin system largely regulates feed intake in animals through melanocortin receptors 3 and 4, and *MC4R *has been shown in rats to increase in levels during fasting [30]. Mutations in *αMSH *or *MC4R *result in obesity in mice [12]. In chickens, administration of αMSH markedly decreases feed intake [31,32]. In contrast to mammals, which only express *MC4R *in the brain, chicken *MC4R *is expressed in the adrenals, gonads, spleen, adipose, and brain [33]. Though hypothalamic *MC4R *mRNA levels were not significantly changed by fasting or feeding in the present experiment, they were reduced significantly in neurons from chicks fed for 48 h and treated with αMSH, NPY, NE, or LSOP. Due to the fact that elevated αMSH levels are associated with satiety (fed chicks) and elevated NPY levels are associated with fasting (fasted chicks), the similar effects of both hormones on *MC4R *expression deserves further consideration. Nonetheless, our findings support an integral role for *MC4R *in controlling metabolism and suggest that regulation of its expression is complex.

We hypothesize that the two receptors within the gene network that function as monoamine neurotransmitter receptors, ADRB2 and GRM8, may function to integrate signals emanating from the melanocortin pathway in the hypothalamus. SRIF, αMSH, and NPY reduced expression of both *ADRB2 *and *GRM8 *in this study. This occurred in fasted groups only for *ADRB2 *and for fed and fasted groups for *GRM8*. Importantly, agonists for both receptors have been associated with feed intake in other studies. In chickens, administration of NE into the ventromedial nucleus, paraventricular nucleus, or medial septal areas of the hypothalamus increased feed intake [34]. We observed decreased *POMC *expression at 24 h and *MC4R *expression at 48 h in fed chicks in response to administration of NE *in vitro*. Decreases in POMC and MC4R are associated with increased appetite [34]. Interestingly, injection of glutamate into the lateral hypothalamus of rats results in increased feed intake [35]. We also observed effects of the GRM8 receptor agonist LSOP on *ADRB2 *expression in neurons from fasted chicks, but *ADRB2 *expression was decreased at 24 h and increased at 48 h. Our results support a role for hypothalamic adrenergic and glutaminergic neurons and ADRB2 and GRM8 in regulating feed intake and metabolism.

The effect of treatments in vitro was dependent on the nutritional state (fed or fasted) of the donor animals. *POMC *was decreased by NE only in neurons from fed chicks. Perhaps this was due to the fact that *POMC *expression is higher in neurons from fed than fasted chicks. Likewise, NPY decreased *GRM8 *expression only in neurons from fed chicks, which may be attributed to the fact that NPY is naturally increased in fasted chicks. Further, *MC4R *was only regulated in neurons from fed chicks, and its expression was consistently decreased by all treatments at 48 h. Additionally, the increase in *NPY *expression in response to αMSH was only observed in neurons from fasted chicks, when *α MSH *expression would be low and *NPY *expression would be high. It should be noted that an observed lack of effect of an agonist does not preclude the possibility that the system is already maximally stimulated. In general, differences in gene expression in response to treatment were more dramatic following 48 h of feeding and fasting than at 24 h. This is expected, due to the fact that the effects of fasting or feeding should be more pronounced after a longer period of time.

Treatment with the hormones and agonists for receptors in the putative gene network resulted in altered expression of other genes in the network. The fact that gene expression responses differed based upon the nutritional status of the chicks provides evidence that this pathway may indeed participate in the regulation of metabolic responses to feeding and fasting. As expected, the melanocortin pathway, involving *POMC *and *MC4R*, appears to play a critical role in feeding and satiety, along with *NPY*, and potentially *AGRP*. As αMSH has been shown in mice to regulate TRH and CRH [36,37], and NPY and AGRP also function to reduce thyroid function [14,38], the interaction of these genes likely influences many aspects of metabolism. The fact that all hormone treatments influenced mRNA levels for the neurotransmitter receptors ADRB2 and GRM8 suggests the possibility that neural coordination of metabolism occurs through these receptors.

## Conclusions

These experiments utilized microarray analysis to evaluate gene expression profiles in the hypothalamus of newly hatched chicks. We found that expression of numerous genes was affected by fasting, and most were returned to the same level as fully fed chicks within 48 h of delayed feeding. A gene network consisting of 6 differentially regulated genes was associated from our list of 119 candidate genes. We confirmed interactions among these genes through culture of primary hypothalamic neurons. Our data indicate that the NPY (NPY, NPYR5) and melanocortin pathways (POMC, MC4R, AGRP) may play an integral part in the regulation of feed intake and metabolism by the hypothalamus and that the receptors ADRB2 and GRM8 may be involved in the regulation or effector mechanisms of these pathways. Further research is needed to completely elucidate potential interactions among these genes.

## Methods

### Animals

All animal experiments were approved by the institutional animal care and use committee at the University of Maryland, College Park. Fertile broiler eggs (Ross × Cobb) were obtained from a local hatchery, and were incubated under standard conditions (37.5 C and 60% relative humidity), with turning every hour for 18 days. On day 18, eggs were transferred to a hatching cabinet and were no longer rotated. Male chicks were identified by feather sexing at hatching, which was confirmed by visual inspection of the gonads at the time of dissection. Chicks receiving feed were fed a commercially available starter diet (Chick Start-N-Grow, CM-25-236007, Cooperative Milling, Gettysburg, PA) *ad libitum*, which was formulated to meet or exceed NRC nutrient recommendations (NRC, 1994). This diet also included the anti-coccidial drug Amprolium at 0.0125%.

### Experiment 1: Fasting and Delayed Feeding

After hatching, male chicks were either provided immediate access to feed *ad libitum *or brooded with no access to feed. Only chicks that hatched within a 3-hour interval, 9:00 am to 12:00 pm on the 21st day of incubation, were used in order to reduce variability in age of post-hatch chicks. Chicks were brooded in cages after hatch beginning at 1pm, and all experimental samples were collected at 1pm on appropriate days. All feed was provided to delayed-fed chicks at 9 am or 1pm, and then chicks were sampled at the appropriate time point (Table 1). All chicks received free access to water and 24 h of light throughout the experiment. Ten groups were included in this experiment (Table 1). At each sampling time, chicks were weighed. After chicks were killed, the yolk sacs were removed and weighed. Hypothalamus samples were carefully dissected from 16 male chicks from each experimental group at the designated time. Initial incisions were made just anterior to the occulomotor nerve (Nervus occulomotorius) and posterior to the Tuberculum olfactorium, based on published descriptions and diagrams [39]. Next, lateral cuts were made approximately 2 mm from the midline to yield a rectangular piece of tissue. This was placed on its side, and a final cut was made at a depth immediately below the subseptal organ (Organum subseptale) and parallel to the basal surface of the hypothalamus. Hypothalami were pooled to reduce the effect of variability among dissections and to ensure sufficient RNA for amplification. Two hypothalami were placed in a single cryotube and immediately snap frozen in liquid nitrogen for subsequent RNA isolation.

### Microarray Analysis

Microarrays consisting of 21,120 oligonucleotides were obtained from the Genomics Research Laboratory at the Steele Children's Research Center at the University of Arizona http://www.grl.steelecenter.arizona.edu/products.asp. This array was developed by ARK-Genomics http://www.ark-genomics.org/microarrays/bySpecies/chicken/ using chicken ENSEMBL transcripts, and covers much of the chicken genome. Annotation of this array is available at GEO http://www.ncbi.nlm.nih.gov/projects/geo/query/acc.cgi?acc=GPL6049. Total cellular RNA was isolated from the hypothalamus using the RNeasy Midi Kit (Qiagen, Valencia, CA) according to manufacturer's protocol. Quantification was accomplished using the Ribogreen assay (Invitrogen), and quality was evaluated by visualizing samples in a formaldehyde gel.

Two μg of pooled total RNA from two samples (4 hypothalami total) were then used for each amplification of mRNA using a modification of the Eberwine procedure [40] through the Amino Allyl MessageAmp™ II aRNA Amplification Kit (Ambion, Austin, TX). Briefly, reverse transcription was performed using an oligo dT primer containing a T7 promoter. The purified cDNA provided a template for *in vitro *transcription, which resulted in antisense amplified copies of mRNA containing the modified nucleotide 5-(3-aminoallyl)-UTP, which is necessary for dye labelling with the Alexa fluor dyes. Samples consisting of 20 μg of amplified RNA (aRNA) were labelled with Alexa fluor dyes (Invitrogen, Carlsbad, CA) and purified. Following analysis with the NanoDrop ND-1000 Full-spectrum UV/Vis Spectrophotometer (Thermo Scientific, Wilmington, DE) to determine concentration, 8 μg of purified labelled aRNA was hybridized to the microarrays using a reference design [41].

An internal reference standard, created by pooling aRNA from all samples within the experiment, was labeled with Alexa Fluor 647. Experimental samples labelled with Alexa Fluor 555 were hybridized to individual microarrays along with the Alexa 647-labeled reference pool. Labeled aRNA samples, plus 2.5 μl of 10 mg/ml yeast tRNA and 2.5 μl of 10 mg/ml salmon testes DNA (Sigma, St. Louis, MO), were hybridized to microarray slides overnight at 42C in microarray hybridization buffer (Roche Diagnostics Corporation, Indianapolis, IN). Following hybridization, slides were washed carefully with increasing stringency using salt sodium citrate and scanned with a 418 confocal laser scanner (Affymetrix) at 555 nm and 647 nm. Two TIFF images were obtained for each slide.

The data were analyzed according to established protocols in our laboratory as previously described [42]. Images were initially analyzed using GenePix Pro 6.0 software (Molecular Devices, Sunnyvale, CA). The numeric data were then exported for data normalization using Microarray Data Analysis System (MIDAS; version 2.18). Data from the Alexa 555 channel (the experimental sample) were Lowess normalized by block without background correction, followed by standard deviation regularization first by block and then by slide, using the Alexa 647-labeled pool as a reference. The log_2 _ratio (normalized Alexa 555/Alexa 647; sample/reference pool) for each spot was calculated.

Prior to statistical analysis, Lowess normalized data were trimmed as follows. First, all results for individual genes on each array returning greater than 90% saturated pixel intensities and pixel intensities less than three times background were eliminated. Second, all genes missing more than 10 datapoints (25% of total) were discounted from further analysis. Third, any gene exhibiting less than a 1.6-fold difference among treatment groups in its mean pixel intensity (0.65 difference in the log_2 _ratios) were likewise eliminated. The resulting trimmed data were analyzed statistically using a one-way ANOVA (SAS) to compare gene expression between treatment groups. Spots (representing 119 genes total) determined to be statistically significant (p < 0.05) among treatment groups were analyzed further. Of the 119 genes, 31 were undescribed, and 12 represented hypothetical proteins. The data from this experiment are deposited in the Gene Expression Omnibus, Accession # GSE13257 http://www.ncbi.nlm.nih.gov/geo/query/acc.cgi?acc=GSE13257.

### Pathway Analysis

Genes that were differentially regulated due to treatment (missing no more than 25% data points, 1.6-fold difference in expression, P < 0.05) were further analyzed using the web-accessible Pathway Miner tool [43], freely available at http://www.biorag.org/pathway.html. This tool is capable of searching three freely available pathway resources: KEGG, Biocarta, and GenMAPP. Pathway Miner determines if there are pathways that are highly represented (enriched) within a dataset. For analysis, human protein ortholog NCBI accession numbers were manually assigned, and were available for 87 of the 119 genes. These 87 genes were submitted for analysis. GO terms were also assigned to these genes, and the biological process GO IDs are available in Additional file 3, supplemental table 3S. Additionally, analysis by Ingenuity Pathway Analysis software http://www.ingenuity.com was performed on two sets of genes. Initial analysis included genes that were differentially regulated only on d1 or d2 between fed and fasted chicks, and a second analysis was performed by submission of all 119 genes differentially expressed in this experiment. The pathways from this analysis are included in additional files 4, 5 and 6.

### qRT-PCR analysis

Quantitative reverse-transcription-real time polymerase chain reaction (qRT-PCR) was performed on 12 genes to confirm gene expression patterns observed by the microarray. All eight individual samples were used for the qRT-PCR analysis, due to the fact that the cost is much less for qRT-PCR than for microarray analysis, and inclusion of more samples in the qRT-PCR assay allowed more accurate analysis of gene expression. Two-step qRT-PCR was performed on all 80 samples. Each reaction included 0.5 μg of total RNA, an oligo dT primer (5'-CGGAATTCTTTTTTTTTTTTTTTTTTTTV-3': Sigma Genosys, St.Louis, MO), Superscript III reverse transcriptase and RNAse Out RNAse inhibitor (Invitrogen, Carlsbad, CA). A negative control for genomic DNA contamination was prepared by pooling RNA from each sample, and using 0.5 μg in a reaction without addition of Superscript III. All first strand cDNA reactions were diluted 5-fold prior to use in PCR reactions.

PCR primers were designed with the Primer 3 program [44], available for use at the URL http://fokker.wi.mit.edu/primer3/input.htm, utilizing the full-length mRNA sequence predicted from the chicken genome available through ENSEMBL http://www.ensembl.org/Gallus_gallus/Search/Results?species=Gallus_gallus;idx=;q=gallus as template. Primer sequences utilized in these experiments are detailed in Table 3. mRNA levels were quantified using the MyiQ Single-Color Real-Time PCR Detection System (Bio-Rad) and the 2× Quantitect SYBR Green PCR Master Mix (Bio-Rad). Cycles were performed as follows: denaturation at 95C for 3 min to activate the polymerase, followed by 40 cycles of 95C for 15 s, 60C for 30 s, and 72C for 30 s. Dissociation curve analysis and gel electrophoresis were utilized to ensure that a single PCR product was amplified in each reaction. Data were normalized to the housekeeping gene *PGK1*, and data were transformed using the equation 2^-Ct^, where Ct represents the fractional cycle number when the amount of amplified product reaches a threshold for fluorescence. Data were divided by the mean of the expression levels at hatch or basal level of gene expression in a given experiment for statistical analysis and comparison with microarray results. Results were then analyzed statistically (ANOVA) to confirm statistically significant effects of treatments as described previously [42].

**Table 3:** Primer sequences utilized for quantitative real-time PCR (qRT-PCR).

RIGG#	Gene Name	ENSEMBL ID (ENSGALG0000_)	Forward Sequence (5'-3')	Reverse Sequence (3'-5')
12704	SSTR5	005258	AACAGCTGTGCCAACCCTAT	CCTCTACACCATTGCCCTTT
15132	NPY5R	09499	TTCCACATTGTGACGGATTT	AGCAGCAGGACATCATACCA
15762	DIO2	10520	ACTGTTTGAGGGCGCTAAACC	AAACACTAGCCCTCCAGAATACCTT
15385	COCH	09920	TAGAGGACCTGCTGTTGCTG	GGTTCAGAGGCCATGTCTTT
17342	CYP19A1	13294	GTGCTTTTGGATGCAGTACC	CCTTTCATTCCCAGCCTTTA
00156	FKBP5	00947	TTTGCCAAGTTTGCTGAGAGG	CCTCTGTCTCTTTGCCTTCATCA
19293	CYP39A1	16710	GTGCTTTTGGATGCAGTACC	CCTTTCATTCCCAGCCTTTA
19221	POMC	16600	CGCTACGGCGGCTTCA	TCTTGTAGGCGCTTTTGACGAT
18192	FABP7	14866	AAATGGGATGGCAAAGAGAC	TTCTCATAGTGGCGAACAGC
16984	SALL3	12657	CACCCATACTGGTGAAAAACC	TTATTCCACATGTGCGTTCC
08723	PKCi	09364	GCAATGTTGATTGGGATCTG	TTGGTGAACTGGGAATCAAA
11322	ADRA2C	02808	CCTTCAACCCCCTGATCTAC	CCGTTGGCATACATCTTCAG
02139	GHRH	03842	AGGAGAAGGGGTGCACAA	CTCCCAAGAAGTCCCTCAGT
14532	TRH	08490	ATGCTTCAATCTGTCCTCAAGA	GTCTCCAAAAGATGCTTTTTCC
17124	MC4R	12907	CGGGAGGCTGCTATGAACAA	AGCTGATGATGCCCAGAGTCA
14136	FGF16	07806	ACGAGAGAGGCGAGCTGTAT	AGAGTGTTGAGGCGTACGTG
13934	HBA1	07468	TGGACCCTGTCAACTTCAAA	GAACTTGTCCAGGGAAGCAT
	CRH	15521	CATCTCCCTGGACCTGACTT	CCATCAGTTTCCTGTTGCTG
	NPY	10983	GGGAAAGCACAGAAAACATTCC	AAATCCCATCACCACATCGAA
	LEPR	11058	TTCCAAACCCCAAGAATTGCT	CAAATGACATTGCTTCAGGGTG
	AGRP	02244	AAGTCTGGCCTGGGAAGAG	CCCCCTGCAGAAGATGAG

### Experiment 2: 24 or 48 h Fasted and Fed

Chicks were hatched as described for Experiment 1, but were maintained in brooder batteries for only 24 or 48 h with or without feed. At the end of 24 or 48 h, the hypothalamus was carefully dissected from 8 chicks each (32 chicks total), with 2 hypothalami pooled per tube (n = 4 pooled samples). Total RNA was extracted using the RNeasy Midi Kit, and reverse transcription followed by qRT-PCR for selected genes was performed as described above.

### Experiment 3: Primary Cell Culture of Hypothalamic Neurons

Chicks were hatched as described above, and male chicks were placed in a brooder battery for 24 or 48 h with or without feed. Hypothalami were dissected from chicks (8 per treatment, per time) and placed in warm Hank's balanced salt solution containing 10 mM Hepes and 0.011 μL/mL sodium pyruvate (HBSS). The hypothalami were sequentially transferred to two additional dishes of warm HBSS using a sterile 5 mL serological pipet, then finally to 10 mL of SMEM medium (with Earle's salts, no L-glutamine, plus 110 U/mL penicillin G and streptomycin and 13.6 μL/mL bovine serum albumen) in a 15 mL conical tube containing 1 mg/mL trypsin. Tissues were incubated at 37C for 1 h, with trituration every 15 minutes to dissociate the cells. Cells were centrifuged and washed twice with DMEM (plus 110 U/mL penicillin G and streptomycin and 13.6 μL/mL bovine serum albumen). Following the last centrifugation, cells were resuspended in 1 mL of Neurobasal Medium containing B-27 supplement and 110 U/mL penicillin G and streptomycin (NBM). Cells were subjected to live/dead staining with trypan blue and enumerated. Six wells per treatment group and time point were plated at a concentration of 1 × 10^6 ^cells/well in poly-L-lysine coated 12-well plates in 100 μL volume. Following incubation at 37C for 1 h, 900 μL of NBM were added to each well, and neurons were incubated overnight. All cell culture media and additives were obtained from Invitrogen (Carlsbad, CA).

Cells were allowed to incubate for a total of 24 h to recover, after which receptor agonists were added in a 10 μL volume. Treatments included the following: PBS (control), alpha-melanocyte stimulating hormone (αMSH) (1 × 10^-7 ^M), neuropeptide Y (NPY) (1 × 10^-7 ^M), somatostatin (SRIF) (1 × 10^-7 ^M), norepinephrine (NE) (1 × 10^-4 ^M), and O-phospho-L-serine (LSOP) (1 × 10^-3 ^M) [45] (all from Sigma-Aldrich, St. Louis, MO). Treatments were incubated in individual wells for 24 h, after which neurons were collected by retrypsinization in SMEM containing 0.25 mg/mL trypsin. RNA was extracted from the cells using the RNeasy Mini Kit (Qiagen, Valencia, CA), and reverse transcription was performed as described above, with the exception that the reaction was accomplished using 50 ng of RNA, and cDNA was diluted 1:1 before use. qRT-PCR for expression of pro-opiomelanocortin (*POMC*), melanocortin receptor 4 (*MC4R*), neuropeptide Y (*NPY*), neuropeptide Y receptor 5 (*NPYR5*), somatostatin receptor 5 (*SSTR5*), beta-adrenergic receptor 2 (*ADRB2*), and glutamate receptor 8 (*GRM8*), as well as the housekeeping gene phosphoglycerate kinase 1 (*PGK1*), was performed using the methods described above.

Statistics were performed on these data initially by use of a 3-way ANOVA comparing age, fed state, and cell culture treatment. As we were able to detect significant differences by both age and feeding state of the chicks, the data were then analyzed by age and treatment within a feeding state (fed or fasted). The data are expressed as relative to the control for each age and feeding state to determine only the effects of the agonists on each gene.

## Authors' contributions

SEH performed experiments 2 and 3, pathway analysis, and drafted the manuscript. LEE performed experiment 1, hybridized, and performed preliminary analysis of the microarray. NT also hybridized and performed preliminary analysis of the microarrays. FM annotated the Gene Ontology terms for the gene list. JS performed qRT-PCR. LAC and TEP conceived the study and assisted in drafting the manuscript. All hypothalamic dissections were performed by TEP. All authors read and approved the final manuscript.

## Supplementary Material

Additional file 1 **Table S1 - Genes that are upregulated in newly hatched chicks by fasting for 48 h as compared to feeding for 48 h**. List of upregulated genes comparing 48 h fasting with fed chicks, contains Genbank accession numbers, RIGG ID, description, and p-values. Click here for file

Additional file 2 **Table S2 - Genes that are downregulated in newly hatched chicks by fasting for 48 h as compared to feeding for 48 h**. List of upregulated genes comparing 48 h fasting with fed chicks, contains Genbank accession numbers, RIGG ID, description, and p-values. Click here for file

Additional file 3 **Table S3 - Biological Process Gene Ontology terms for genes differentially regulated in the microarray analysis**. List of differentially regulated genes, contains Genbank accession number, RIGG ID, biological process GO ID number and description. Click here for file

Additional file 4 **Figure S1.** Gene Network #1 from differentially regulated genes between Fed and Fasted chicks on D1 and D2 (p < 0.05, Fold Difference ≥ 1.4). Gene network diagram. Click here for file

Additional file 5 **Figure S2.** Gene Network #2 from differentially regulated genes between Fed and Fasted chicks on D1 and D2 (p < 0.05, Fold Difference ≥ 1.4). Gene network diagram. Click here for file

Additional file 6 **Figure S3.** Gene Network #3 from all differentially regulated genes in the Hypothalamus (p < 0.05, Fold Difference ≥ 1.6). Gene network diagram. Click here for file
