# Supplementary material for: Transcriptional and pathway analysis in the hypothalamus of newly hatched chicks during fasting and delayed feeding
Source: BMC Genomics. 2010 Mar 9;11:162. doi: 10.1186/1471-2164-11-162 (PMC2848243; doi:10.1186/1471-2164-11-162)
Supplement: Additional file 1 — Table S1 - Genes that are upregulated in newly hatched chicks by fasting for 48 h as compared to feeding for 48 h. List of upregulated genes comparing 48 h fasting with fed chicks, contains Genbank accession numbers, RIGG ID, description, and p-values. [file 1471-2164-11-162-S1.DOC]

Table S1. Genes upregulated in newly hatched chicks by fasting for 48 h as compared with feeding for 48h.

| **Genbank**  **Accession** | **RIGG ID**  **(RIGG_)** | **Description** | **Trt.**  **Prob.** | **Age**  **Prob.** | **Inter.**  **Prob.** | **Group.**  **Prob.** |
| --- | --- | --- | --- | --- | --- | --- |
| CR406142 | 04436 | _ | 0.000 | 0.007 | 0.025 | 0.000 |
| CR733141 | 17342 | Cytochrome P450, family 19, subfamily A, polypeptide 1 | 0.000 | 0.003 | 0.014 | 0.000 |
| NW_001471613 | 00156 | FK-506 binding protein 51 | 0.000 | 0.001 | 0.006 | 0.000 |
| BX934029 | 09007 | Hypothetical protein LOC772201 | 0.002 | 0.000 | 0.019 | 0.000 |
| CR353193 | 15385 | Coagulation factor C homolog, cochlin | 0.040 | 0.000 | 0.125 | 0.002 |
| XM_420063 | 04151 | _ | 0.000 | 0.005 | 0.102 | 0.000 |
| XM_421662 | 11954 | Syndecan 4_Syndecan-4 precursor | 0.004 | 0.086 | 0.044 | 0.002 |
| XM_417138 | 09431 | Sestrin-1 (p53-regulated protein PA26) | 0.000 | 0.000 | 0.011 | 0.000 |
| XM_414883 | 02842 | _ | 0.001 | 0.672 | 0.006 | 0.000 |
| CR353208 | 08446 | Ectonucleoside triphosphate diphosphohydrolase 1 | 0.025 | 0.374 | 0.282 | 0.185 |
| XM_421662 | 02033 | ATG10 autophagy related 10 homolog (S. cerevisiae)_ | 0.003 | 0.118 | 0.227 | 0.018 |
| NW_001471556 | 12925 | Similar to LOC495522 protein | 0.000 | 0.009 | 0.079 | 0.000 |
| XM_416561 | 01071 | _ | 0.000 | 0.937 | 0.114 | 0.000 |
| CR352990 | 04276 | _ | 0.027 | 0.422 | 0.014 | 0.007 |
| AF304358 | 04517 | _ | 0.011 | 0.036 | 0.290 | 0.056 |
| CR390842 | 08884 | Tetratricopeptide repeat protein 8 | 0.040 | 0.023 | 0.812 | 0.063 |
| XM_416405 | 09940 | Similar to class I alpha chain | 0.035 | 0.010 | 0.207 | 0.035 |
| AJ719645 | 09819 | Growth arrest and DNA-damage-inducible, gamma | 0.007 | 0.257 | 0.475 | 0.004 |
| CR389786 | 08177 | Similar to protein tyrosine phosphatase domain containing 1 protein | 0.001 | 0.003 | 0.104 | 0.000 |
| XM_419178 | 19211 | Similar to hypothetical protein | 0.035 | 0.631 | 0.035 | 0.064 |
| AJ719912 | 16531 | _ | 0.094 | 0.556 | 0.011 | 0.009 |
| CR523366 | 04740 | _ | 0.005 | 0.099 | 0.832 | 0.056 |
| BX934863 | 09403 | Phosphatidylinositol-4-phosphate 5-kinase, type I, beta | 0.092 | 0.234 | 0.028 | 0.036 |
| NW_001471543 | 15762 | Deiodinase, iodothyronine, type II | 0.001 | 0.418 | 0.565 | 0.000 |
| BX935773 | 08290 | Similar to histidine decarboxylase | 0.015 | 0.000 | 0.473 | 0.000 |
| BX932384 | 18298 | Relaxin 3 | 0.017 | 0.019 | 0.698 | 0.004 |
| X59541 | 11740 | Vitronectin | 0.041 | 0.011 | 0.093 | 0.000 |
| XM_418968 | 00847 | Glutamine synthetase | 0.006 | 0.112 | 0.725 | 0.003 |
| CR389657 | 19390 | _ | 0.030 | 0.067 | 0.400 | 0.003 |
| XM_415051 | 19058 | Hypothetical LOC421890 | 0.019 | 0.935 | 0.932 | 0.159 |
| BX930144 | 11157 | Regulator of G-protein signaling 2 | 0.001 | 0.005 | 0.166 | 0.000 |
| CR407576 | 12677 | Aquaporin-1 | 0.017 | 0.217 | 0.158 | 0.118 |
| XM_423753 | 15132 | Neuropeptide Y receptor Y5 | 0.022 | 0.003 | 0.722 | 0.000 |
| CR524143 | 13315 | D-2-hydroxyglutarate dehydrogenase | 0.018 | 0.131 | 0.431 | 0.113 |
| NW_001471594 | 16466 | Matrix Gla-protein precursor | 0.026 | 0.022 | 0.382 | 0.141 |
| NW_001471446 | 03982 | Similar to Neuronal pentraxin II precursor | 0.041 | 0.004 | 0.641 | 0.000 |
| CR390405 | 11322 | Similar to beta-2 adrenergic receptor | 0.012 | 0.102 | 0.095 | 0.000 |
| XM_001234513 | 10122 | Similar to aminoacylase family member | 0.010 | 0.418 | 0.731 | 0.032 |
| XM_417956 | 01642 | Similar to vitelliform macular dystrophy 2-like 2 | 0.047 | 0.367 | 0.112 | 0.043 |
| AF119370 | 11695 | Myeloblastosis oncogene-like 2 | 0.007 | 0.134 | 0.049 | 0.047 |
| AJ719927 | 08482 | COX15 homolog isoform 1 precursor | 0.000 | 0.668 | 0.623 | 0.018 |
| NM_001006685 | 18245 | Similar to Chromosome 6 open reading frame 113 | 0.246 | 0.292 | 0.012 | 0.069 |
| BX931455 | 12704 | Somatostatin receptor 5 | 0.985 | 0.213 | 0.035 | 0.058 |
| BX929782 | 04171 | Nth endonuclease III-like 1 (E. coli) | 0.356 | 0.516 | 0.045 | 0.254 |
| CR390155 | 09167 | Hypothetical LOC418131 | 0.106 | 0.064 | 0.031 | 0.019 |
| AJ719295 | 04950 | Armadillo repeat containing 7 | 0.018 | 0.119 | 0.164 | 0.031 |
| CR524015 | 03669 | Carboxypeptidase M | 0.011 | 0.094 | 0.788 | 0.111 |
| CR387700 | 14671 | Glutamate receptor, metabotropic 8 precursor | 0.028 | 0.002 | 0.907 | 0.072 |
| XM_423496 | 18223 | Follistatin precursor (FS) | 0.001 | 0.000 | 0.376 | 0.000 |
| AJ851536 | 08380 | Fibroblast growth factor 13 isoform 1s | 0.609 | 0.052 | 0.048 | 0.023 |
| CR387623 | 09352 | Regulator of G-protein signalling 7 binding protein | 0.006 | 0.296 | 0.960 | 0.201 |
| XM_421809 | 20254 | Suppressor of var1, 3-like 1 (S. cerevisiae) | 0.692 | 0.314 | 0.019 | 0.037 |
| XM_426579 | 07647 | Similar to glial fibrillary acidic protein alpha | 0.616 | 0.339 | 0.007 | 0.045 |
| U35815 | 08438 | SAPS domain family, member 3 | 0.748 | 0.758 | 0.027 | 0.230 |
| CR339034 | 08741 | YY1 associated factor 2 | 0.026 | 0.553 | 0.432 | 0.018 |
| BX933155 | 13978 | Adenomatosis polyposis coli down-regulated 1-like | 0.006 | 0.222 | 0.150 | 0.354 |
| NW_001471575 | 20415 | Similar to decay-accelerating factor (GDab-TCS) | 0.951 | 0.752 | 0.035 | 0.291 |
| BX935931 | 13082 | Solute carrier organic anion transporter family, member 5A1 | 0.003 | 0.055 | 0.292 | 0.006 |
| BX935704 | 15397 | Solute carrier family 35, member E3 | 0.014 | 0.970 | 0.894 | 0.245 |
| CR352348 | 04551 | Bcl-2 inhibitor of transcription | 0.047 | 0.434 | 0.022 | 0.025 |
