# Supplementary material for: Transcriptional and pathway analysis in the hypothalamus of newly hatched chicks during fasting and delayed feeding
Source: BMC Genomics. 2010 Mar 9;11:162. doi: 10.1186/1471-2164-11-162 (PMC2848243; doi:10.1186/1471-2164-11-162)
Supplement: Additional file 2 — Table S2 - Genes that are downregulated in newly hatched chicks by fasting for 48 h as compared to feeding for 48 h. List of upregulated genes comparing 48 h fasting with fed chicks, contains Genbank accession numbers, RIGG ID, description, and p-values. [file 1471-2164-11-162-S2.DOC]

Table S2. Genes downregulated in newly hatched chicks by fasting for 48 h as compared with feeding for 48h.

| **Genbank**  **Accession** | **RIGG ID**  **(RIGG_)** | **Description** | **Trt.**  **Prob.** | **Age**  **Prob.** | **Inter.**  **Prob.** | **Group**  **Prob.** |
| --- | --- | --- | --- | --- | --- | --- |
| CR388587 | 02675 | _ | 0.042 | 0.219 | 0.945 | 0.097 |
| XM_424329 | 10043 | Hypothetical LOC426707_ | 0.023 | 0.094 | 0.245 | 0.115 |
| XM_421330 | 08903 | Similar to LOC495018 protein | 0.017 | 0.820 | 0.445 | 0.042 |
| XM_417138 | 19544 | _ | 0.024 | 0.324 | 0.183 | 0.035 |
| CR389341 | 03898 | _ | 0.023 | 0.141 | 0.585 | 0.142 |
| BX932757 | 03282 | Uncharacterized glycosyltransferase AGO61 precursor | 0.032 | 0.718 | 0.696 | 0.471 |
| AJ720854 | 14143 | NmrA-like family domain containing 1 | 0.165 | 0.713 | 0.008 | 0.168 |
| CR388665 | 02771 | Hypothetical protein | 0.016 | 0.022 | 0.085 | 0.004 |
| U65891 | 17219 | Protein tyrosine phosphatase, receptor type, O_ | 0.049 | 0.769 | 0.666 | 0.443 |
| CR523039 | 03964 | _ | 0.029 | 0.002 | 0.832 | 0.001 |
| NW_001471543 | 11649 | _ | 0.024 | 0.048 | 0.461 | 0.097 |
| XM_421854 | 11255 | Nucleoporin 35kDa_ | 0.002 | 0.029 | 0.701 | 0.021 |
| XM_421847 | 07878 | Collagen alpha-1(III) chain precursor | 0.002 | 0.033 | 0.313 | 0.004 |
| BX930280 | 10682 | Musculoskeletal, embryonic nuclear protein 1 | 0.027 | 0.000 | 0.027 | 0.000 |
| CR388915 | 19596 | Similar to RIKEN cDNA 2810485I05 | 0.350 | 0.057 | 0.025 | 0.011 |
| AJ295030 | 17212 | Aldo-keto reductase family 1, member B10 (aldose reductase) | 0.009 | 0.490 | 0.311 | 0.052 |
| NW_001471449 | 07349 | _ | 0.493 | 0.026 | 0.031 | 0.066 |
| CR523332 | 12378 | _ | 0.007 | 0.002 | 0.346 | 0.003 |
| CR352883 | 02848 | Hypothetical protein\ | 0.041 | 0.622 | 0.172 | 0.162 |
| XM_417573 | 10575 | ATPase family, AAA domain containing 3B | 0.019 | 0.009 | 0.103 | 0.017 |
| AF193760 | 00003 | SRY (sex determining region Y)-box 14 | 0.015 | 0.439 | 0.886 | 0.358 |
| BX932607 | 10408 | Runt-related transcription factor | 0.036 | 0.011 | 0.258 | 0.000 |
| BX932987 | 06541 | Formin-2 | 0.049 | 0.060 | 0.165 | 0.023 |
| BX931741 | 19005 | Similar to RIKEN cDNA 4930578C19_ | 0.008 | 0.465 | 0.129 | 0.077 |
| BX933964 | 08295 | RUN and FYVE domain containing 1_heterogeneous nuclear ribonucleoprotein H1 | 0.022 | 0.138 | 0.918 | 0.083 |
| XR_027084 | 12681 | Similar to Dgat2l1-prov protein | 0.032 | 0.647 | 0.985 | 0.317 |
| XM_425989 | 15951 | Similar to Fer3-like | 0.006 | 0.128 | 0.375 | 0.025 |
| NM_001006685 | 16449 | Heat shock 70 kDa protein | 0.007 | 0.460 | 0.883 | 0.120 |
| AJ720043 | 13755 | Small nuclear ribonucleoprotein polypeptide A | 0.036 | 0.363 | 0.610 | 0.371 |
| CR389501 | 07146 | _ | 0.010 | 0.002 | 0.516 | 0.001 |
| XM_419178 | 09413 | Hypothetical LOC421093 | 0.013 | 0.148 | 0.815 | 0.352 |
| V00390 | 07369 | Collagen, type I, alpha 2 | 0.006 | 0.012 | 0.185 | 0.001 |
| BX935301 | 06883 | _ | 0.037 | 0.594 | 0.869 | 0.031 |
| BX931258 | 04879 | _ | 0.037 | 0.001 | 0.169 | 0.001 |
| NW_001471556 | 07428 | Serpin H1 precursor (Collagen-binding protein) | 0.016 | 0.432 | 0.537 | 0.039 |
| CR353204 | 01052 | _ | 0.032 | 0.531 | 0.559 | 0.458 |
| XM_414136 | 08131 | Similar to system asc amino acid transporter Asc-1 | 0.038 | 0.002 | 0.005 | 0.001 |
| AF133251 | 19071 | Glutathione S-transferase A3 | 0.026 | 0.002 | 0.169 | 0.003 |
| M60657 | 18189 | 3-hydroxy-3-methylglutaryl coenzyme A synthase | 0.026 | 0.617 | 0.827 | 0.035 |
| AJ719295 | 19715 | Insulin induced gene 1_Sonic hedgehog protein precursor (SHH) | 0.030 | 0.241 | 0.952 | 0.006 |
| NW_001471707 | 07298 | _ | 0.038 | 0.385 | 0.270 | 0.272 |
| XR_027074 | 09397 | Similar to protein tyrosine phosphatase, receptor type, D | 0.033 | 0.204 | 0.767 | 0.010 |
| L16955 | 17263 | Islet amyloid polypeptide | 0.666 | 0.009 | 0.005 | 0.008 |
| BX935733 | 13636 | _ | 0.035 | 0.139 | 0.235 | 0.102 |
| X00169 | 16779 | Histone H5 | 0.000 | 0.066 | 0.029 | 0.000 |
| CR386400 | 09835 | Hypothetical protein | 0.013 | 0.283 | 0.467 | 0.086 |
| CR352419 | 17857 | _ | 0.250 | 0.378 | 0.004 | 0.031 |
| CR390522 | 17776 | Arginine vasopressin | 0.047 | 0.001 | 0.704 | 0.018 |
| CR389164 | 01697 | Cell division control protein 2 homolog (Cyclin-dependent kinase 1) | 0.016 | 0.001 | 0.308 | 0.000 |
| XR_027119 | 09962 | Similar to importin beta subunit | 0.047 | 0.688 | 0.437 | 0.244 |
| NM_001031098 | 19221 | Proopiomelanocortin | 0.000 | 0.001 | 0.600 | 0.000 |
| CR389737 | 03278 | _ | 0.007 | 0.184 | 0.610 | 0.082 |
| CR523285 | 04320 | _ | 0.016 | 0.001 | 0.021 | 0.000 |
| XM_420063 | 19293 | Similar to Cyp39a1 protein | 0.030 | 0.156 | 0.995 | 0.473 |
| AJ720450 | 00240 | Sterile alpha motif domain containing 11 | 0.016 | 0.571 | 0.298 | 0.143 |
| CR523193 | 04483 | _ | 0.005 | 0.321 | 0.025 | 0.001 |
| AJ851678 | 08723 | Similar to Protein kinase C, iota type | 0.045 | 0.247 | 0.045 | 0.085 |
| AF304358 | 16984 | Sal-like 3 (Drosophila) | 0.024 | 0.648 | 0.564 | 0.432 |
| X65459 | 18192 | Fatty acid binding protein 7, brain | 0.001 | 0.022 | 0.095 | 0.000 |
