# Supplementary material for: Transcriptional and pathway analysis in the hypothalamus of newly hatched chicks during fasting and delayed feeding
Source: BMC Genomics. 2010 Mar 9;11:162. doi: 10.1186/1471-2164-11-162 (PMC2848243; doi:10.1186/1471-2164-11-162)
Supplement: Additional file 3 — Table S3 - Biological Process Gene Ontology terms for genes differentially regulated in the microarray analysis. List of differentially regulated genes, contains Genbank accession number, RIGG ID, biological process GO ID number and description. [file 1471-2164-11-162-S3.DOC]

Table S3. Biological Process Gene Ontology terms for genes differentially regulated in the microarray analysis.

| **Genbank Accession** | **RIGG#** | **Gene Name** | **BP GO ID** | **BP GO Term Name** |
| --- | --- | --- | --- | --- |
| CR523202 | 04436 | Gallus gallus finished cDNA, clone ChEST629c13 | 0003674 | molecular_function |
| CR523193 | 04483 | Gallus gallus finished cDNA, clone ChEST634d9 | 0008150 | biological_process |
| U65891 | 17219 | Protein tyrosine phosphatase C | 0006470  0016311 | protein amino acid dephosphorylation  dephosphorylation |
| CR353204 | 01052 | Gallus gallus finished cDNA, clone ChEST140e9 | 0008150 | biological_process |
| XM_425195 | 11322 | Similar to beta-2 adrenergic receptor | 0007186  0002032  0006898  0007190  0043410  0001993  0002024  0002025  0002028  0007189  0009409  0030501  0031649  0040015  0045453  0045944  0045986  0050873 | G-protein coupled receptor protein signaling pathway  desensitization of G-protein coupled receptor protein signaling pathway by arrestin  receptor-mediated endocytosis  activation of adenylate cyclase activity  positive regulation of MAPKKK cascade  regulation of systemic arterial blood pressure by norepinephrine-epinephrine  diet induced thermogenesis  vasodilation by norepinephrine-epinephrine involved in regulation of systemic arterial blood pressure  regulation of sodium ion transport  G-protein signaling, adenylate cyclase activating pathway  response to cold  positive regulation of bone mineralization  heat generation  negative regulation of multicellular organism growth  bone resorption  positive regulation of transcription from RNA polymerase II promoter  negative regulation of smooth muscle contraction  brown fat cell differentiation |
| XM_413833 | 08290 | Similar to histidine decarboxylase | 0006519  0019752  0006548 | cellular amino acid and derivative metabolic process  carboxylic acid metabolic process  histidine catabolic process |
| CR386400 | 09835 | StAR-related lipid transfer (START) domain containing 4 | 0003674 | molecular_function |
| XM_001234723 | 09007 | Hypothetical protein | 0006520 | amino acid metabolic process |
| XM_417573 | 10575 | Similar to ATAD3A protein |  |  |
| AF133251 | 19071 | Glutathione S-transferase clas | 0008152 | metabolic process |
| NW_001471737 | 11157 | RGS2: Regulator of G-protein signaling 2 |  |  |
| NW_001471449 | 07349 | BR serine/threonine kinase 2 | 0006468  0030182  0030010 | protein amino acid phosphorylation  neuron differentiation  establishment of cell polarity |
| BX932866 | 02033 | Hypothetical protein | 0006497  0031401 | protein amino acid lipidation  positive regulation of protein modification process |
| XM_425989 | 15951 | Similar to Fer3-like | 0045449 | regulation of transcription |
| XM_421311 | 08884 | Tetratricopeptide repeat domain 8 | 0001947  0046907  0051877 | heart looping  intracellular transport  pigment granule aggregation in cell center |
| AJ851429 | 08438 | SAPS domain family, member 3 |  |  |
| XM_426640 | 12925 | Similar to LOC495522 protein | 0007596 | blood coagulation |
| M60657 | 18189 | Hydroxymethylglutaryl-CoA Synthase | 0006084  0006694  0006695  0008610  0016126 | acetyl-CoA metabolic process  steroid biosynthetic process  cholesterol biosynthetic process  lipid biosynthetic process  sterol biosynthetic process |
| CR353438 | 01071 | Hypothetical protein LOC769345 | 0008150 | biological_process |
| XM_419178 | 09413 | Hypothetical protein | 0007186  0007601 | G-protein coupled receptor protein signaling pathway  visual perception |
| Y11030 | 11740 | Vitronectin | 0007155  0006955  0007160 | cell adhesion  immune response  cell-matrix adhesion |
| CR386157 | 13978 | Adenomatosis polyposis coli down-regulated 1-like | 0008150 | biological_process |
| NW_001471512 | 14671 | Similar to metabotropic glutamate receptor 8 | 0007601  0007268  0030818  0035249  0007268 | visual perception  synaptic transmission  negative regulation of cAMP biosynthetic process  "synaptic transmission, glutamatergic"  synaptic transmission |
| XM_419796 | 09431 | Sestrin 1 | 0006974  0007050  0008285 | response to DNA damage stimulus  cell cycle arrest  negative regulation of cell proliferation |
| AJ720854 | 14143 | NmrA-like family domain containing 1 | 0006808 | regulation of nitrogen utilization |
| NW_001471707 | 07298 | Similar to MGC82377 protein |  |  |
| CR523285 | 04320 | PREDICTED: hypothetical protein XP_430095 | 0008150 | biological_process |
| BX930280 | 10682 | Musculoskeletal, embryonic nuclear protein 1 | 0003674 | molecular_function |
| AJ720043 | 13755 | Small nuclear ribonucleoprotein polypeptide A |  |  |
| BX935733 | 13636 | Cyclin J | 0000074 | regulation of progression through cell cycle |
| CR390800 | 00847 | Glutamine synthetase | 0006542  0006807 | glutamine biosynthetic process  nitrogen compound metabolic process |
| XM_416363 | 09167 | Tyrosyl-tRNA synthetase 2, mitochondrial | 0006412  0006418  0006437 | Translation  tRNA aminoacylation for protein translation  tyrosyl-tRNA aminoacylation |
| AY954512 | 12704 | Somatostatin receptor type 5 | 0007165  0007186  0007218 | signal transduction  G-protein coupled receptor protein signaling pathway  neuropeptide signaling pathway |
| XR_027074 | 09397 |  |  |  |
| AJ720354 | 20254 | RCJMB04_16a1: Hypothetical protein |  |  |
| L16955 | 17263 | Amyloid protein precursor |  |  |
| BX931258 | 04879 | CDC28 protein kinase regulatory subunit 2 | 0000079  0007049  0007051  0008283  0048015  0000910  0007049  0007127 | regulation of cyclin-dependent protein kinase activity  cell cycle  spindle organization and biogenesis  cell proliferation  phosphoinositide-mediated signaling  cytokinesis  cell cycle  meiosis I |
| XM_001232965 | 09819 | Growth arrest and DNA-damage-inducible, gamma | 0006281  0006950  0000185  0000074  0045063  0006469  0042095  0000186 | DNA repair  response to stress  activation of MAPKKK activity  regulation of progression through cell cycle  T-helper 1 cell differentiation  negative regulation of protein kinase activity  interferon-gamma biosynthetic process  activation of MAPKK activity |
| CR388587 | 02675 | Prenylcysteine oxidase 1 like | 0030328 | prenylcysteine catabolic process |
| NW_001471556 | 07428 | Serpin H1 precursor | 0006457 | protein folding |
| CR389501 | 07146 | Ectodysplasin A1 | 0006817  0006955 | phosphate transport  immune response |
| V00390 | 07369 | Collagen, type I, alpha 2 | 0006817 | phosphate transport |
| XR_027084 | 12681 |  |  |  |
| XM_421330 | 08903 | Chromogranin A precursor | 0008217 | blood pressure regulation |
| BX936132 | 18298 | Relaxin 3 | 0008150 | biological_process |
| AF108755 | 08380 | Fibroblast growth factor 13 | 0007165  0007267  0007399 | signal transduction  cell-cell signaling  nervous system development |
| CR352419 | 17857 | LOC420048 | 0008150 | biological_process |
| BX932005 | 01642 | Similar to vitelliform macular dystrophy 2-like 2 | 0008150 | biological_process |
| CR389341 | 03898 | Gallus gallus finished cDNA, clone ChEST559i23 | 0008150 | biological_process |
| CR354188 | 13315 | D-2-hydroxyglutarate dehydrogenase | 0006118  0010042  0010043  0032025  0032026  0044267  0051592 | electron transport  response to manganese ion  response to zinc ion  response to cobalt ion  response to magnesium ion  cellular protein metabolic process  response to calcium ion |
| BX932074 | 19390 | Hypothetical LOC418773 | 0008150 | biological_process |
| XM_424329 | 10043 | Hypothetical LOC426707 | 0016180 | snRNA processing |
| BX929861 | 08446 | Ectonucleoside triphosphate diphosphohydrolase 1_ |  |  |
| BX932987 | 06541 | Similar to formin 2 | 0016043  0030036 | cellular component organization  actin cytoskeleton organization |
| XM_417138 | 19544 | SACS, KIAA0730: Sacsin | 0006457  0006464 | protein folding  protein modification process |
| CR388473 | 04151 | Similar to LOC387763 protein | 0008150 | biological_process |
| CR388915 | 19596 | Similar to RIKEN cDNA 2810485I05 | 0008150 | biological_process |
| NM_001031098 | 19221 | Pro-opiomelanocortin |  |  |
| CR389184 | 15132 | Neuropeptide Y receptor Y5 | 0007165  0007186  0007218 | signal transduction  G-protein coupled receptor protein signaling pathway  neuropeptide signaling pathway |
| BX950696 | 04950 | Armadillo repeat containing 7 | 0008150 | biological_process |
| BX935129 | 03669 | Carboxypeptidase M | 0009653 | anatomical structure morphogenesis |
| AF193760 | 00003 | SRY (sex determining region Y)-box 14 | 0006350  0006355 | Transcription  "regulation of transcription, DNA-dependent" |
| AJ720576 | 09403 | Phosphatidylinositol-4-phosphate 5-kinase, type I, beta | 0046488 | phosphatidylinositol metabolic process |
| AJ851678 | 08723 | Similar to Protein kinase C, iota type | 0006468  0007242 | protein amino acid phosphorylation  intracellular signaling cascade |
| BX932607 | 10408 | Hypothetical protein LOC777379 | 0008150 | biological_process |
| BX932757 | 03282 | Uncharacterized glycosyltransferase AGO61 precursor |  |  |
| AY723747 | 00156 | FKBP51: FK-506 binding protein | 0006457 | protein folding |
| BX935301 | 06883 | Similar to NPD014 protein (NPD014) | 0008150 | biological_process |
| CR388665 | 02771 | Gallus gallus finished cDNA, clone ChEST386m17 | 0006461  0006886  0016192 | protein complex assembly  intracellular protein transport  vesicle-mediated transport |
| XR_027119 | 09962 |  |  |  |
| NW_001471633 | 12677 | Aquaporin-1 | 0006810 | Transport |
| XM_419771 | 18245 | Similar to Chromosome 6 open reading frame 113 | 0008150 | biological_process |
| XM_418091 | 07647 | Similar to glial fibrillary acidic protein alpha |  |  |
| X65459 | 18192 | Fatty acid binding protein 7, brain | 0006810 | Transport |
| NW_001471710 | 15762 | Type II iodothyronine deiodinase | 0006590  0042446 | thyroid hormone generation  hormone biosynthetic process |
| NW_001471543 | 11649 | Midline 1 |  |  |
| NM_001007869 | 11649 | Similar to WWC family member 3 |  |  |
| XM_418287 | 11649 | Similar to chloride channel protein 4 | 0006821  0006810 | chloride transport  transport |
| AJ719916 | 11649 | Hypothetical LOC418648 | 0008150 | biological_process |
| AF012252 | 11954 | Syndecan-4 precursor |  |  |
| XM_421854 | 13082 | Solute carrier organic anion transporter family, member 5A1 | 0006810 | Transport |
| X67505 | 10122 | Similar to aminoacylase family member | 0006508 | proteolysis |
| CR385374 | 15385 | Cochlin precursor | 0007605 | sensory perception of sound |
| AJ720159 | 11255 | Nucleoporin 35kDa | 0006605  0006810  0015031  0051028 | protein targeting  transport  protein transport  mRNA transport |
| AJ295030 | 11695 | Myb-related protein B | 0006350  0006355  0045449 | Transcription  "regulation of transcription, DNA-dependent"  regulation of transcription |
| CR406003 | 04551 | Peptidyl-tRNA hydrolase 2 | 0008150 | biological_process |
| AY675346 | 08741 | YY1 associated factor 2 | 0016481  0045941 | negative regulation of transcription  positive regulation of transcription |
| XM_421700 | 17212 | Aldo-keto reductase family 1, member B10 |  |  |
| CR389038 | 15397 | Solute carrier family 35, member E3 | 0008150 | biological_process |
| CR523039 | 16531 | Exosome component 9_Polymyositis/scleroderma autoantigen 1 | 0006396 | RNA processing |
| NM_001006685 | 08482 | COX15 homolog, cytochrome c oxidase assembly protein (yeast) | 0006118  0006123  0006461  0006784  0007585  0008535  0045333 | electron transport  "mitochondrial electron transport, cytochrome c to oxygen"  protein complex assembly  heme a biosynthetic process  respiratory gaseous exchange  cytochrome c oxidase complex assembly  cellular respiration |
| XM_414322 | 02842 | Hypothetical protein LOC769739 | 0008150 | biological_process |
| AJ719295 | 03964 | Chromogranin A (parathyroid secretory protein 1) | 0008217  0006996 | blood pressure regulation  organelle organization and biogenesis |
| NW_001471479 | 16449 | Heat shock 70 kDa protein | 0009408 | response to heat |
| AJ720450 | 08177 | Similar to protein tyrosine phosphatase domain containing 1 protein | 0006470 | protein amino acid dephosphorylation |
| CR352883 | 19715 | Insulin induced gene 1 | 0008283  0008152  0006991 | cell proliferation  metabolic process  response to sterol depletion |
| BX950566 | 09940 | Similar to class I alpha chain | 0006955  0019882  0006955 | immune response  antigen processing and presentation  immune response |
| CR390522 | 00240 | Sterile alpha motif domain containing 11 | 0000122 | negative regulation of transcription from RNA polymerase II promoter |
| X87609 | 02848 | WDR68 WD repeat domain 68 | 0051216 | cartilage development |
| NW_001471513 | 03982 | Neuronal pentraxin II | 0007268 | synaptic transmission |
| CR523332 | 17776 | Vasotocin-neurophysin VT precursor |  |  |
| XM_001234191 | 18223 | Follistatin precursor | 0000122  0007276  0007389  0008585  0030509  0042475  0045596  0007275  0046882 | negative regulation of transcription from RNA polymerase II promoter  gamete generation  pattern specification process  female gonad development  BMP signaling pathway  odontogenesis (sensu Vertebrata)  negative regulation of cell differentiation  multicellular organismal development  negative regulation of follicle-stimulating hormone secretion |
| BX933964 | 16466 | Matrix Gla-protein precur | 0001503  0007275  0030154  0030500  0051216 | ossification  multicellular organismal development  cell differentiation  regulation of bone mineralization  cartilage development |
| AF304358 | 12378 | Similar to bA9F11.1 | 0008150 | biological_process |
| J04047 | 09352 | Hypothetical protein LOC777379 | 0008150 | biological_process |
| XM_421847 | 08295 | RUN and FYVE domain containing 1 | 0030100  0015031 | regulation of endocytosis  protein transport |
| BX930531 | 16984 | Spalt 2 protein |  |  |
| CR391469 | 17342 | Cytochrome P450, family 19, subfamily A, polypeptide 1 | 0006118  0006118 | electron transport  electron transport |
| XM_416834 | 07878 | Collagen alpha-1(III) | 0006817 | phosphate transport |
| X00169 | 04171 | Nth endonuclease III-like 1 (E. coli) | 0006281  0006284 | DNA repair  base-excision repair |
| BX931741 | 04517 | Tectonic family member 2 | 0008150 | biological_process |
| XM_429893 | 19211 | Similar to hypothetical protein |  |  |
| CR523932 | 16779 | Histone H5 | 0006334  0007001  0007076 | nucleosome assembly  chromosome organization and biogenesis (sensu Eukaryota)  mitotic chromosome condensation |
| XM_414136 | 19005 | Similar to RIKEN cDNA 4930578C19 | 0008150 | biological_process |
| BX934543 | 19058 | Hypothetical LOC421890 | 0008150 | biological_process |
| XM_420063 | 04740 |  |  |  |
| XM_417981 | 08131 | Similar to system asc amino acid transporter Asc-1 | 0006810  0006865  0015804 | transport  amino acid transport  neutral amino acid transport |
| CR389737 | 04276 | Cysteine-rich protein 2 | 0008284  0030097 | positive regulation of cell proliferation  hemopoiesis |
| CR389164 | 19293 | Similar to Cyp39a1 protein | 0006118  0006699  0006707 | electron transport  bile acid biosynthetic process  cholesterol catabolic process |
| CR523202 | 20415 | Complement decay accelerating factor | 0006955  0006958  0045087 | immune response  "complement activation, classical pathway"  innate immune response |
| CR523193 | 03278 | Gallus gallus finished cDNA, clone ChEST472b7 | 0008150 | biological_process |
| U65891 | 01697 | Cell division control protein 2 | 0006468  0007049  0007067  0007095  0051301 | protein amino acid phosphorylation  cell cycle  mitosis  mitotic G2 checkpoint  cell division |
