# Supplementary figures and images for: Transcriptional and pathway analysis in the hypothalamus of newly hatched chicks during fasting and delayed feeding
Source: BMC Genomics. 2010 Mar 9;11:162. doi: 10.1186/1471-2164-11-162 (PMC2848243; doi:10.1186/1471-2164-11-162)

## Slide 1
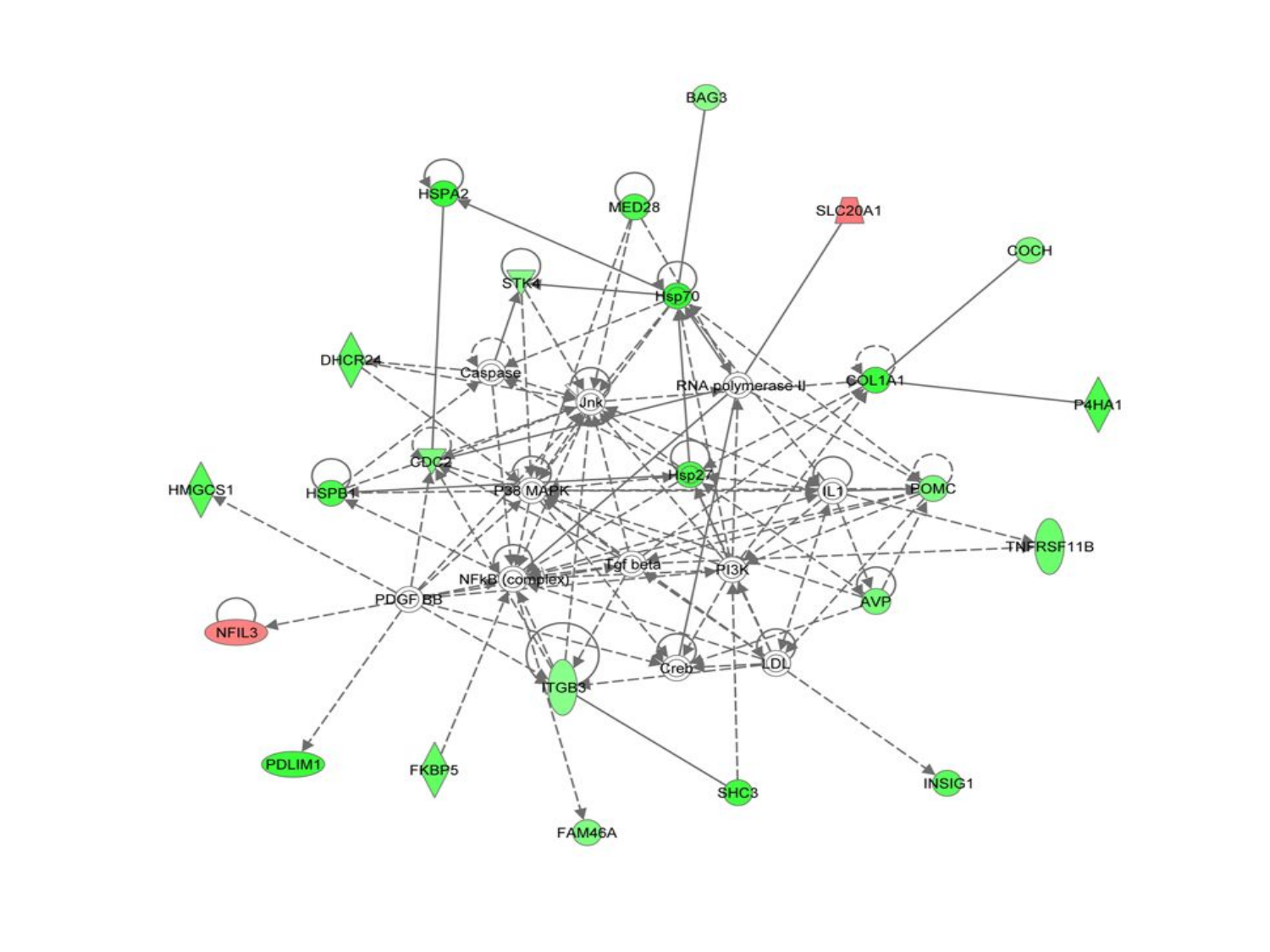

Supplement: Additional file 4 — Figure S1. Gene Network #1 from differentially regulated genes between Fed and Fasted chicks on D1 and D2 (p < 0.05, Fold Difference ≥ 1.4). Gene network diagram. [file 1471-2164-11-162-S4.PPTX]

## Slide 1
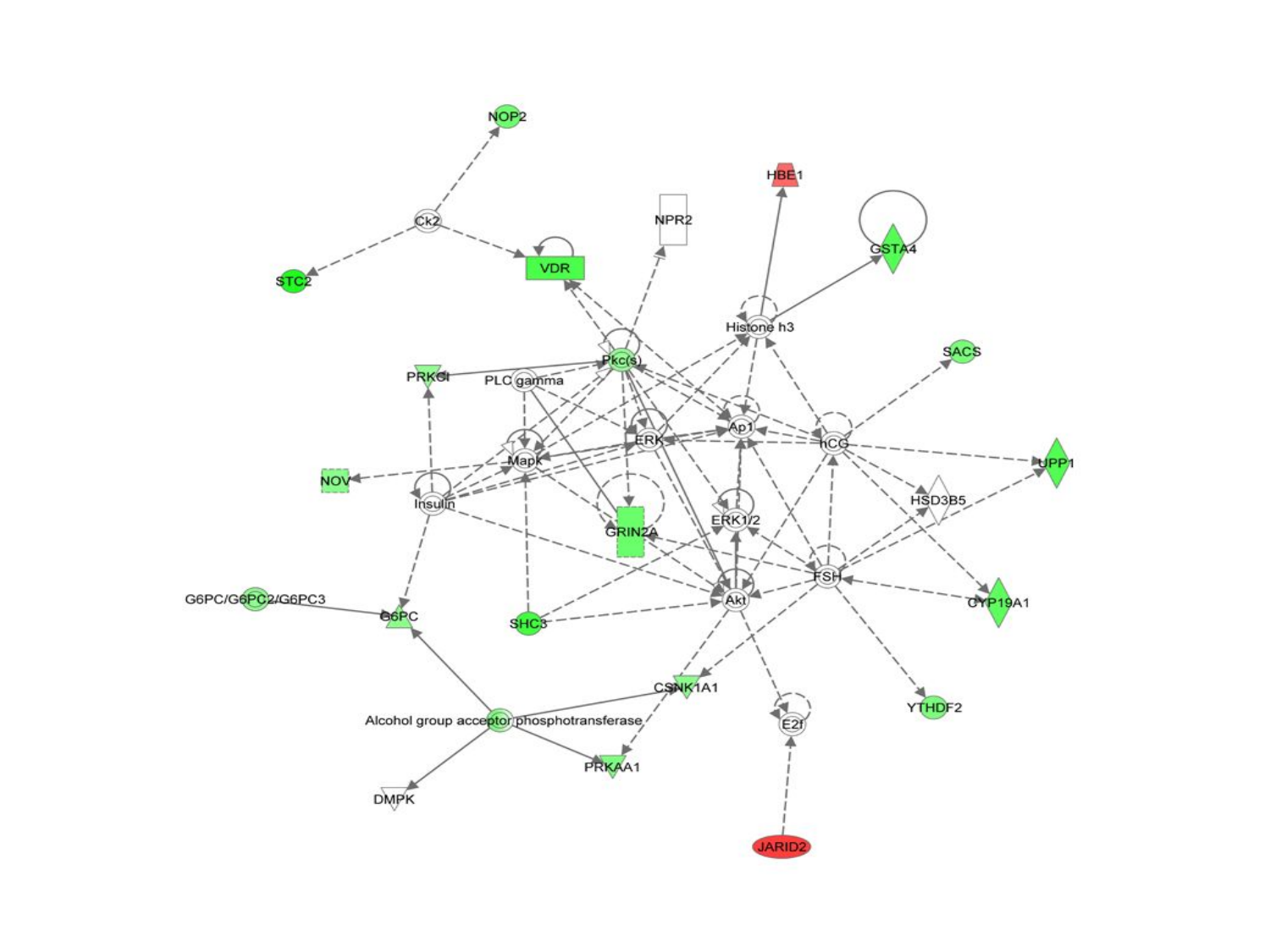

Supplement: Additional file 5 — Figure S2. Gene Network #2 from differentially regulated genes between Fed and Fasted chicks on D1 and D2 (p < 0.05, Fold Difference ≥ 1.4). Gene network diagram. [file 1471-2164-11-162-S5.PPTX]

## Slide 1
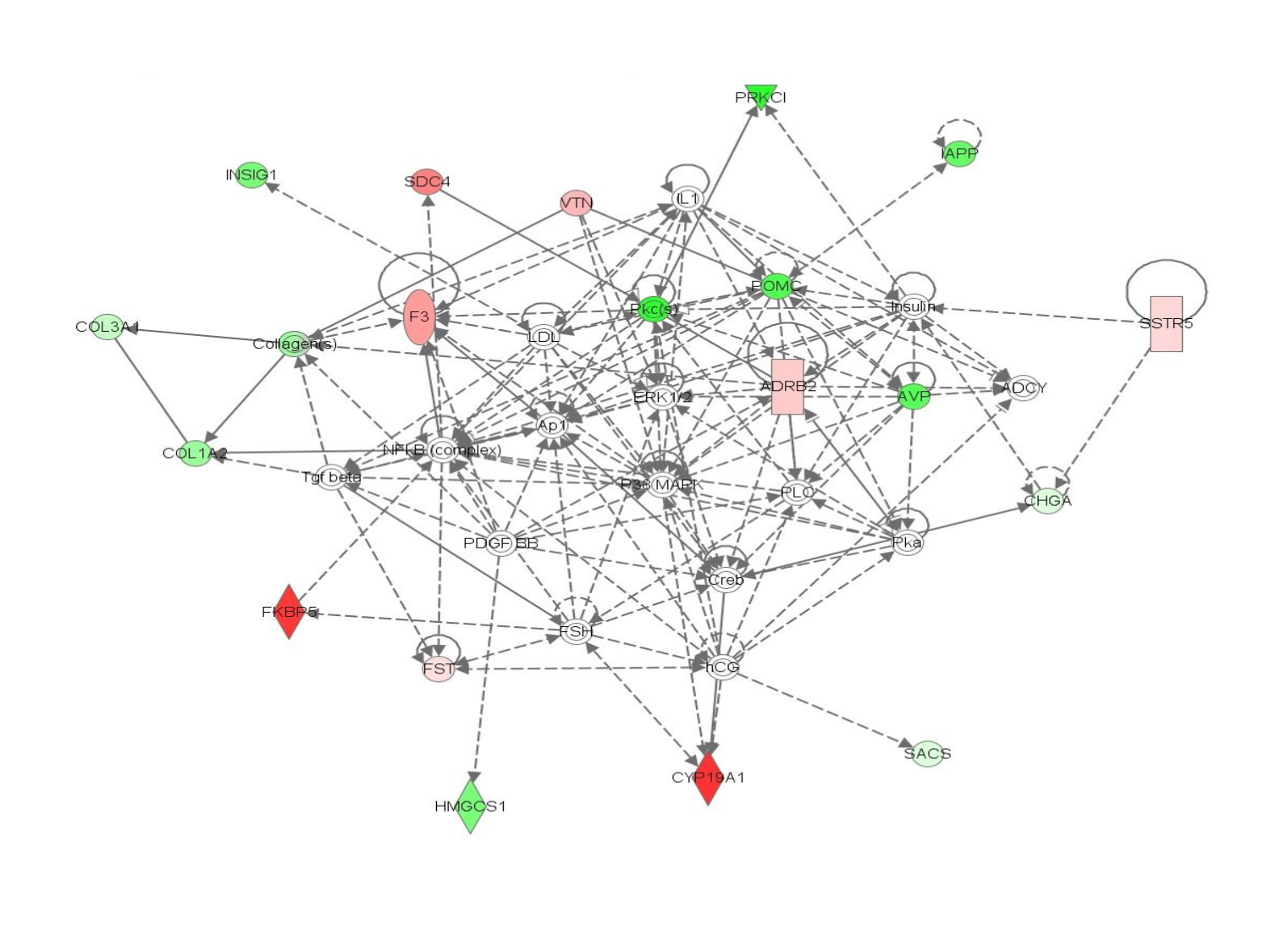

Supplement: Additional file 6 — Figure S3. Gene Network #3 from all differentially regulated genes in the Hypothalamus (p < 0.05, Fold Difference ≥ 1.6). Gene network diagram. [file 1471-2164-11-162-S6.PPTX]
